# Supplementary material for: Factors influencing the functionality of medicines and therapeutic committees in public health facilities in Uganda: A longitudinal assessment
Source: PLoS One. 2026 Feb 11;21(2):e0342487. doi: 10.1371/journal.pone.0342487 (PMC12893557; doi:10.1371/journal.pone.0342487)
Supplement: S1 File — (PDF) [file pone.0342487.s002.pdf]

FUNCTIONALITY OF MEDICINES AND THERAPEUTIC COMMITTEES IN PUBLIC HEALTH FACILITIES IN UGANDA: A LONGITUDINAL ASSESSMENT

SURVEY QUESTIONNAIRE

Introduction: This is to introduce you to an assessment study of the current functionality of MTCs in public health facilities. The study is intended to establish the state and functionality of MTCs and guide any further interventions.

Confidentiality: *Seek Informed Consent.* Your responses shall be treated as confidential and will not be presented anywhere individually but rather as an aggregated summary of findings. Kindly spare five minutes to read and understand the study objectives and purpose for which the results shall serve. **Interviewer:** Have you understood what the study is all about? **Interviewee:** Yes/No; **Interviewer:** Can we proceed with the interview? **Interviewee:** Yes/No; **Interviewer:** IF YES, may you spare about 60 minutes to respondent to the questions I'm going to ask you? **Interviewee:** Yes/No.
Interviewer: IF YES, let us start the interview.

**SECTION A. BACKGROUND INFORMATION** *Circle the correct code or fill in the response. Codes and numerical responses to be written again in column three.*		Write the codes in the un-shaded box
1.	Questionnaire Number:	
2.	Name of interviewer	
3.	Date of interview (dd/mm/yyyy): ...../....... /.......	
4.	NMS Region:	
5.	Name of the responding officer:.................................	
6.	Title of the responding officer:<br>1=Hospital Director/Medical superintendent/HCIV In-charge<br>2=Pharmacist<br>3=Senior Dispenser/Dispenser<br>4=MTC Chairperson<br>5=MTC Member<br>6=Assistant Inventory Management Officer<br>7=Other (specify)	
7.	How long have you served in this hospital?<br>1=Less than 1 year<br>2= 1 to 2 years<br>3= 3 to 5 years<br>5= 6 years and above	
8.	Hospital name:	
9.	Level of care:<br>1=NRH<br>2=NRI<br>3=RRH<br>4=GH<br>5=HCIV	

<table>
<tr><td>10.</td><td>Nature of the Hospital:
1=Teaching Hospital
2= Non-Teaching Hospital</td><td></td></tr>
<tr><td colspan="2">SECTION B. MTC STRUCTURE Circle the correct code or fill in the response. Codes and numerical responses to be written again in column three.</td><td>Write the codes in the un-shaded box</td></tr>
<tr><td>1.</td><td>Do you have a functional MTC in this hospital?
1= Yes, fully functional
2=Yes, partially functional
3=Yes, but not functional
4=No</td><td>IF No and Qn2 and END</td></tr>
<tr><td>2.</td><td>If No above, why does your hospital lack a functional MTC?
1=Lack of financial support to operationalize the MTC
2=Knowledge gap in MTC operations
3=Lack of commitment from members
4=Lack of will and support from hospital administration
5=Other specify</td><td></td></tr>
<tr><td>3.</td><td>Does the hospital MTC have officially appointed members?
1=Yes, all members are officially appointed
2=Yes, some members are officially appointed
3=No</td><td></td></tr>
<tr><td>4.</td><td>How many members does your MTC have including the Chair and the Secretary? ……………………..</td><td></td></tr>
<tr><td>5.</td><td>Does your hospital have a copy of the following guidelines? Tick all that apply
1=MTC guidelines
2=Pharmacovigilance guidelines
3=Antimicrobial stewardship guidelines
4=National standard treatment guidelines
5=Essential medicine and health supplies list</td><td></td></tr>
<tr><td>6.</td><td>Does your MTC have clear Terms of Reference?
1=Yes
2=No</td><td></td></tr>
<tr><td>7.</td><td>Does your hospital have the following MTC sub-Committees?
<table>
<tr><td>Sub-committee</td><td colspan="4">Functionality level</td></tr>
<tr><td></td><td>1=Very Functional</td><td>2=Partially functional</td><td>available</td><td>Not in place</td></tr>
<tr><td>Pharmacovigilance</td><td></td><td></td><td></td><td></td></tr>
<tr><td>Antimicrobial stewardship</td><td></td><td></td><td></td><td></td></tr>
<tr><td>Supply chain/Logistics</td><td></td><td></td><td></td><td></td></tr>
<tr><td>Research committee</td><td></td><td></td><td></td><td></td></tr>
</table></td><td></td></tr>
</table>

8.	If your MTC lack any of the above committees, what could be the reasons? ***Tick all that apply***<br>1=Lack of financial support to operationalize the MTC sub-committees<br>2=Knowledge gap about MTC sub-committee operations<br>3=Lack of commitment from hospital technical staff to become members of these sub-committees<br>4=Lack of will and support from hospital administration<br>5=Other specify	
**SECTION C: MTC OPERATIONS**<br>*Circle the correct code or fill in the response. Codes and numerical responses to be written again in column three.*		Write the codes in the un-shaded box
1.	Does your MTC have a work plan, and a budget included in the hospital workplan?<br>1=MTC has a work plan and a budget<br>2=MTC has a work plan but without a budget<br>3=No	
2.	IF a budget is available, what is the source of funding? ***Tick all that apply***<br>1=The hospital<br>2=IPs<br>3=Both the hospital and IPs<br>3=MoH<br>4=others, Specify	
3.	IF your hospital receives MTC external support, what kind of support does it receive? ***Tick all that apply***<br>1=Capacity building such as trainings/mentorship<br>2=Financial support<br>3=Both Capacity building such as trainings and financial support<br>4=Other specify………………………	
4.	If your MTC receives any support from IPs, which IPs support your MTC activities?<br>1=…………………………………<br>2=…………………………………<br>3=…………………………………<br>4=…………………………………	
5.	How often does the hospital MTC meet?<br>1=Every month<br>2=Once in every two months<br>3=Quarterly<br>4=Once in 6 months<br>5=As and when need arises<br>6=Very rare<br>7=Not at all	
6.	Do you have copies of the MTC meeting minutes?<br>1=Yes<br>2=No	
7.	Has your MTC developed any of the following policies and procedures? ***Tick all that apply***	

	1=Management of donations<br>2=Regulation of pharmaceutical promotion<br>3=In-patient pharmacy<br>4=Tracking of medicines and supplies<br>5=Selection, quantification, procurement planning<br>6=Storage, distribution and re-distribution<br>7=Prescription, dispensing, administration of medicines	
8.	Is the hospital MTC involved in the selection/needs analysis of EMHS list?<br>1=Yes, always<br>2=Yes, sometimes<br>3=No	
9.	Is the hospital MTC involved in planning for EMHS during procurement planning and quantification?<br>1=Yes, always<br>2=Yes, sometimes<br>3=No	
10.	Is the hospital MTC involved in, reviewing orders and oversight of EMHS use?<br>1=Yes, always<br>2=Yes, sometimes<br>3=No	
11.	Does the hospital MTC produce regular reports on availability, stock outs, expiries, stock status?<br>1=Yes, always<br>2=Yes, sometimes<br>3=No	
12.	Has your MTC developed an Institutional EMHS list?<br>1=Yes, and updated regularly<br>2=Yes, but not updated<br>2=No	
13.	Has your MTC developed a facility-based antibiogram to guide antibiotic selection?<br>1=Yes, and updated regularly<br>2=Yes, but not updated<br>2=No	
**SECTION D: MEDICINES USE STEWARDSHIP**<br>*Circle the correct code or fill in the response. Codes and numerical responses to be written again in column three.*		Write the codes in the un-shaded box

<table>
<tr><td>1.</td><td>Have you conducted any surveys in the following areas in the last five years?
<table><tr><td rowspan="2">Investigation type</td><td colspan="3">Frequency</td></tr><tr><td>1=Yes, regular (yearly)</td><td>2= Irregular</td><td>No</td></tr><tr><td>overuse of injectable medicines and antibiotics in OPD</td><td></td><td></td><td></td></tr><tr><td>recording of administered medicines in wards</td><td></td><td></td><td></td></tr><tr><td>overstock and understock of certain items</td><td></td><td></td><td></td></tr></table></td><td></td></tr>
<tr><td>2.</td><td>Do you send ADR reports and medicine quality issues to NDA?
1=Yes, as MTC
2=Yes, as a pharmacist
3=Sometimes as MTC, sometimes as a pharmacist
4=No</td><td></td></tr>
<tr><td>3.</td><td>Does your MTC make interventions on CMEs and mentoring in the following areas?
1= Advise medical, pharmacy and administrative staff on appropriate medicine use
2= Restrictions (withdrawal of injectables from OPD)
3= Conduct pharmacovigilance activities
4= Monitor the use of standard treatment guidelines
5= Identify medicines use problems
6= Design and implement antimicrobial stewardship activities
7= Conduct appropriate research on medicine use
8=None</td><td></td></tr>
<tr><td>4.</td><td>Does your hospital MTC monitor prescriptions at OPD?
1=Yes always
2=Yes sometimes
3=No</td><td></td></tr>
<tr><td>5.</td><td>Does your hospital MTC monitor adherence to STG for specific conditions
1=Yes always
2=Yes sometimes
3=No</td><td></td></tr>
<tr><td>6.</td><td>Does your hospital MTC monitor changes and adherence to prescription practices targeting specific interventions (as per THE problem identified)?
1=Yes
2=No</td><td></td></tr>
</table>

<table>
<tr><td></td><td></td><td></td></tr>
<tr><td colspan="2">SECTION E: OVERALL PERFORMANCE AND RECOMMENDATIONA
Circle the correct code or fill in the response. Codes and numerical responses to be written again in column three.</td><td>Write the number in each unshaded box</td></tr>
<tr><td>1.</td><td>On a scale of 1 to 10, how would you rate the performance of your MTC in performing its functions of; evaluating and improving the clinical use of EMHS, developing and/or monitoring policies and procedures and managing an institutional medicine list? Rank on a scale of 1-10
<table><tr><td rowspan="2">Rank</td><td>1</td><td>2</td><td>3</td><td>4</td><td>5</td><td>6</td><td>7</td><td>8</td><td>9</td><td>10</td></tr><tr><td></td><td></td><td></td><td></td><td></td><td></td><td></td><td></td><td></td><td></td></tr></table></td><td></td></tr>
<tr><td>2.</td><td>If your MTC is operational, what should be done to improve and strengthen its performance?
…………………………………………………………………
…………………………………………………………………
…………………………………………………………………
…………………………………………………………………</td><td></td></tr>
<tr><td>3.</td><td>If your MTC is not functional, what should be done to fully operationalize it?
…………………………………………………………………
…………………………………………………………………
…………………………………………………………………
…………………………………………………………………</td><td></td></tr>
<tr><td></td><td></td><td></td></tr>
</table>

END

THANK YOU
