# Supplementary material for: Factors influencing the functionality of medicines and therapeutic committees in public health facilities in Uganda: A longitudinal assessment
Source: PLoS One. 2026 Feb 11;21(2):e0342487. doi: 10.1371/journal.pone.0342487 (PMC12893557; doi:10.1371/journal.pone.0342487)
Supplement: Attachment — Submitted filename: PONE-D-25-14259_reviewer.pdf [file pone.0342487.s004.pdf]

PLOS ONE

Factors influencing the Functionality of Medicine and Therapeutics Committees in Public Health Facilities: A longitudinal assessment

--Manuscript Draft--

**Manuscript Number:**	PONE-D-25-14259
**Article Type:**	Research Article
**Full Title:**	Factors influencing the Functionality of Medicine and Therapeutics Committees in Public Health Facilities: A longitudinal assessment
**Short Title:**	Functionality of Medicine and Therapeutics Committees in Public Health Facilities: A longitudinal assessment
**Corresponding Author:**	Benard Nsubuga, Masters<br>National medical Stores, Kampala, Uganda<br>Entebbe, UGANDA
**Keywords:**	Functionality, medicines, therapeutics committees, public health facilities, panel data
**Abstract:**	Background: Medicines use problems have become more prevalent in recent times. World health organization estimates that globally, irrational medicine use may be about fifty percent, mostly attributed to overuse of antibiotics, indiscriminate use of injectables, and polypharmacy tendencies. Globally, medicines and therapeutic committees (MTCs) play a significant role in combating inappropriate medicines use problems. This study explored the extent of the functionality of MTCs in health facilities in Uganda and the associated factors.<br>Methodology: This was a longitudinal assessment study. Panel data analysis techniques were used to compare results between the two waves. This study was quantitative in nature and stochastic model estimation processes utilizing the ordered logistic regression model and random effects techniques were used to determine the factors influencing the functionality of MTCs in HFs.<br>Findings: Overall, the percentage of health facilities with a functional MTC significantly increased from 8.3% (22/264) in wave one to 20.8% (55/265) in wave two (p-value<0.05). The percentage of MTCs that are partially functioning increased from 15.2% (40/264) in wave one to 29.1% (77/265) in wave two. The percentage of HFs with MTC structures in place but not carrying out MTC operations increased from 2.6% in wave one to 42.6% in wave two. Health facilities without an MTC in place reduced from 72% (190/264) in wave one to 7.6% (20/265) in wave two (p-value<0.05). MTCs that regularly conduct surveys on overuse of injectable medicines in outpatient departments reduced from 16.2% in wave one to only 7.8% in wave two. Overall, the predicted probability of having fully functional MTCs is 25%. Statistically, level of care (p-value<0.05), availability of guidelines (p-value<0.05), availability of subcommittees (p-value<0.05), and MTC members being active (p-value<0.05) were significantly associated with the performance of MTCs.<br>Conclusions: The study established a significant improvement in the prevalence and functionality of MTCs in health facilities attributed to interventions from national medical stores, Uganda's ministry of health and implementing partners. The study observed little improvement in the MTC operations, especially in carrying out activities that drive the fight against inappropriate use of medicines. Interventions in medication use stewardship should be strengthened at all levels of care.
**Order of Authors:**	Benard Nsubuga, Masters
	Anthony Ddamba, Masters
	Harriet Akello, Masters
	David Arinaitwe, Masters
	Phillip Ampaire, Masters
	Moses Kamabare, Masters
**Additional Information:**	
**Question**	**Response**

Powered by Editorial Manager® and ProduXion Manager® from Aries Systems Corporation

**Financial Disclosure**<br><br>Enter a financial disclosure statement that describes the sources of funding for the work included in this submission. Review the submission guidelines for detailed requirements. View published research articles from *PLOS ONE* for specific examples.<br><br>This statement is required for submission and **will appear in the published article** if the submission is accepted. Please make sure it is accurate.<br><br>**Funded studies**<br>Enter a statement with the following details:<br>• Initials of the authors who received each award<br>• Grant numbers awarded to each author<br>• The full name of each funder<br>• URL of each funder website<br>• Did the sponsors or funders play any role in the study design, data collection and analysis, decision to publish, or preparation of the manuscript?<br><br>Did you receive funding for this work?	The author(s) received no specific funding for this work.
**Competing Interests**<br><br>Use the instructions below to enter a competing interest statement for this submission. On behalf of all authors, disclose any competing interests that could be perceived to bias this work—acknowledging all financial support and any other relevant financial or non-financial competing interests.<br><br>This statement is required for submission and **will appear in the published article** if the submission is accepted. Please make sure it is accurate and that any funding sources listed in your Funding Information later in the submission form are also declared in your Financial Disclosure statement.	The authors have declared that no competing interests exist.

Powered by Editorial Manager® and ProduXion Manager® from Aries Systems Corporation

View published research articles from *PLOS ONE* for specific examples.<br><br>**NO authors have competing interests**<br><br>Enter: *The authors have declared that no competing interests exist.*<br><br>**Authors with competing interests**<br><br>Enter competing interest details beginning with this statement:<br><br>*I have read the journal's policy and the authors of this manuscript have the following competing interests: [insert competing interests here]*<br><br>* typeset	
**Ethics Statement**<br><br>Enter an ethics statement for this submission. This statement is required if the study involved:<br><br>• Human participants<br>• Human specimens or tissue<br>• Vertebrate animals or cephalopods<br>• Vertebrate embryos or tissues<br>• Field research<br><br>Write "N/A" if the submission does not require an ethics statement.<br><br>General guidance is provided below. Consult the submission guidelines for detailed instructions. **Make sure that all information entered here is included in the Methods section of the manuscript.**	The study was conducted in accordance with the principles of the Helsinki Declaration. All participants provided written informed consent, and the study was approved by the research and ethics committee (REC) of Makerere University School of Health Sciences with reference number MAKSHSREC-2024-768 The study was further approved by the Uganda National Council for Science and Technology, under approval number HS5391ES. At all levels, confidentiality was maintained intentionally to protect respondents who volunteered to provide information pertinent to the study. The interviewers introduced the study to the participants, explaining the purpose of the study and the intended benefits. The participants were given five minutes to clearly understand the study and consent to participate.<br><br>The interview process began only after written informed consent had been given by the participant.

Powered by Editorial Manager® and ProduXion Manager® from Aries Systems Corporation

**Format for specific study types**<br><br>**Human Subject Research (involving human participants and/or tissue)**<br>• Give the name of the institutional review board or ethics committee that approved the study<br>• Include the approval number and/or a statement indicating approval of this research<br>• Indicate the form of consent obtained (written/oral) or the reason that consent was not obtained (e.g. the data were analyzed anonymously)<br><br>**Animal Research (involving vertebrate animals, embryos or tissues)**<br>• Provide the name of the Institutional Animal Care and Use Committee (IACUC) or other relevant ethics board that reviewed the study protocol, and indicate whether they approved this research or granted a formal waiver of ethical approval<br>• Include an approval number if one was obtained<br>• If the study involved *non-human primates*, add *additional details* about animal welfare and steps taken to ameliorate suffering<br>• If anesthesia, euthanasia, or any kind of animal sacrifice is part of the study, include briefly which substances and/or methods were applied<br><br>**Field Research**<br><br>Include the following details if this study involves the collection of plant, animal, or other materials from a natural setting:<br>• Field permit number<br>• Name of the institution or relevant body that granted permission	
**Data Availability**<br><br>Authors are required to make all data underlying the findings described fully available, without restriction, and from the time of publication. PLOS allows rare exceptions to address legal and ethical concerns. See the PLOS Data Policy and FAQ for detailed information.	Yes - all data are fully available without restriction

A Data Availability Statement describing where the data can be found is required at submission. Your answers to this question constitute the Data Availability Statement and **will be published in the article**, if accepted.<br><br>**Important:** Stating 'data available on request from the author' is not sufficient. If your data are only available upon request, select 'No' for the first question and explain your exceptional situation in the text box.<br><br>Do the authors confirm that all data underlying the findings described in their manuscript are fully available without restriction?	
**Describe where the data may be found in full sentences. If you are copying our sample text, replace any instances of XXX with the appropriate details.**<br><br>• If the data are **held or will be held in a public repository**, include URLs, accession numbers or DOIs. If this information will only be available after acceptance, indicate this by ticking the box below. For example: *All XXX files are available from the XXX database (accession number(s) XXX, XXX.*).<br>• If the data are all contained **within the manuscript and/or Supporting Information files**, enter the following: *All relevant data are within the manuscript and its Supporting Information files.*<br>• If neither of these applies but you are able to provide **details of access elsewhere**, with or without limitations, please do so. For example:<br><br>*Data cannot be shared publicly because of [XXX]. Data are available from the XXX Institutional Data Access / Ethics Committee (contact via XXX) for researchers who meet the criteria for access to confidential data.*<br><br>*The data underlying the results presented in the study are available from (include the name of the third party*	All relevant data are within the manuscript and its Supporting Information files.

and contact information or URL).<br>• This text is appropriate if the data are owned by a third party and authors do not have permission to share the data.<br><br>* typeset	
Additional data availability information:	

Powered by Editorial Manager® and ProduXion Manager® from Aries Systems Corporation

Manuscript
Click here to access/download;Manuscript;MTC-longitudinal assessment Manuscript v1.docx

Factors Influencing the Functionality of Medicines and Therapeutic Committees in Public Health Facilities in Uganda: A Longitudinal Assessment

Benard Nsubuga
National Medical Stores, Entebbe, Uganda
Anthony Damba
National Medical Stores, Entebbe, Uganda
Harriet Akello
Ministry of Health, Uganda, Department of Pharmaceuticals and Natural Medicines, Kampala, Uganda
David Arinaitwe
National Medical Stores, Entebbe, Uganda
Phillip Ampaire
National Medical Stores, Entebbe, Uganda
Moses Kamabare
National Medical Stores, Entebbe, Uganda

Correspondence: Benard Nsubuga, *bnsubuga22@gmail.com*

Abstract
World health organization estimates that globally, irrational medicine use may be about fifty percent, mostly attributed to overuse of antibiotics and indiscriminate use of injectables, among other things. Globally, medicines and therapeutic committees (MTCs) play a significant role in combating inappropriate medicines use problems. This study explored the extent of the functionality of MTCs in health facilities in Uganda and the associated factors. This was a longitudinal assessment that utilised a semistructured questionnaire that was administered through face-to-face interviews. Panel data analysis techniques were used to compare results between the two waves. This study utilized the ordered logistic regression model and random effects techniques to determine the factors influencing the functionality of MTCs. Overall, the percentage of health facilities with a functional MTC significantly increased from 8.3% (22/264) in wave one to 20.8% (55/265) in wave two (p-value<0.05). The percentage of MTCs that are partially functioning increased from 15.2% (40/264) in wave one to 29.1% (77/265) in wave two. The percentage of HFs with MTC structures increased from 2.6% to 42.6% in wave two. Health facilities without an MTC reduced from 72% (190/264) in wave one to 7.6% (20/265) in wave two (p-value<0.05). MTCs that conducted surveys on overuse of injectable medicines in outpatient departments reduced from 16.2% in wave one to only 7.8% in wave two. Level of care, availability of guidelines, availability of subcommittees, and MTC members being active were significantly associated with the performance of MTCs (p-value<0.05). The study established a significant improvement in the functionality of MTCs in health facilities attributed to interventions from national medical stores, Uganda's ministry of health and implementing partners. The study, however, observed little improvement in MTC operations that drive the fight against inappropriate use of medicines. Interventions in medication use stewardship should be strengthened at all levels of care.

Keywords: Functionality, medicines, therapeutics committees, public health facilities, panel data.

1.0. Background

Medicines use problems have become more prevalent in recent times. World health organization (WHO) estimates that globally, irrational medicines use may be about fifty percent [1] and is more pronounced in the elderly, mostly attributed to polypharmacy tendencies [2,3]. The problems of irrational drug use are mirrored through poor prescribing tendencies, dispensing, and poor patient adherence to use of prescribed medicines [3]. The consequences of inappropriate medicines use range from misuse of scarce resources to adverse therapies clinical consequences and, any intervention intended to promote the rational use of medicines should be integral to the health care management systems. Successful health care systems across the world primarily depend on a myriad of factors including the attributes of leaders governing such health care service delivery points. Globally, medicines and therapeutic committees (MTCs) play a significant role in ensuring appropriate medicines use. MTCs are

the operational arm of health facilities (HFs) intended to improve the efficient management and use of essential medicines. The WHO recognizes the need to conduct assessments on the performance and impact of MTCs as this provides a framework for being self-critical while soliciting for both internal and external support towards MTC operations [4]. MTCs were identified by the WHO as pivotal models for anchoring the rational use of medicines in hospitals at all levels, by providing the leadership and structure needed to identify and choose the right medicines for inclusion into the hospital formulary [5]. This study therefore aimed to establish the current functioning of MTCs and the extent to which they are carrying out structured and targeted interventions within the health facilities to drive the fight against medicine use problems in public health facilities in Uganda.

MTCs are governance bodies aimed at ensuring optimal quality of health care in the population through providing stewardship for cost-effective use of medicines, thereby preventing insufficient therapeutic effect, adverse drug reactions, preventable side-effects and interactions from medicines, and increasing resistance of bacterial pathogens to antimicrobial medicines [4,6]. This is achieved through performing functions such as advisory committee to medical staff, pharmacy, and administration; development of policies and procedures; development of HF formulary list of medicines; develop standard treatment guidelines; assess medicines use problems; conduct effective interventions to improve medicines use problems; and to manage medication errors [4,5]. MTC composition involves a multidisciplinary integration of professionals involved in health care provision to the population. These may include infectious disease specialists, paediatricians, pharmacologists, nurses, pharmacists, representatives of the HF management, public health physician, a general practitioner among others [6].

The presence of MTCs considerably varies between developed and developing economies [7]. Their existence in developed countries dates to eight decades in time and their composition and roles nearly exhibit the same picture in many developed countries [8]. Literature indicates that

the first hospital MTC, formally known as the Drug and Therapeutic Committee (DTC), was formed in 1935. In England, the formulation of DTCs began in the early 1940s, their first hospital MTC was constituted in 1948 and by 1968, nearly seventy five percent of such committees dealt with matters relating to pharmacy work [7,9–11]. To the present, world over, the activities of MTCs include policy formulation, regulation, educational as well as ensuring rational medicines use, contrary to this, at the inception of MTCs in many developed countries, they most focused on establishing drug formularies to meet special requirements of drug availability and dispensing [9].

The composition of many MTCs in developing countries have pharmacists as the most prevalent cadre with an average of two to three pharmacists per hospital MTC, especially in such hospitals with huge bed capacity in at least 95% of hospitals, followed by internal medicine specialist or physicians, and nurses often have the lowest presence [7]. The functioning of any health care facility is directly proportional to the availability of medicines. Patients usually perceive and attribute the quality of health care services offered to medicines accessibility and affordability [12,13]. Not surprisingly, the concept of MTCs in the previous decades mainly revolved around hospital pharmacies, spearheaded by pharmacists and this in part, explains why pharmacists used to be a dominant cadre in the MTC arrangement. Secondly, the availability of medicines does not lie in isolation from their use. In the fight against irrational medicine use, physicians are very pivotal as these not only advocate for patients' responsibility in medicine use but also purpose to enhance the patients understanding of their treatment as well as prevention of any adverse medicine use events.

MTCs are described as a tool designed to advance the improvement of medicine selection, procurement, promote good prescribing and dispensing practices, draft and implement policies and procedures meant to improve use of medicines in health care systems [14]. However, their presence and functionality in low- and middle-income countries (LMICs) is ambivalently

described whose shortcomings manifest from poor funding, poor communication among members, lack of established standards, overburdened committee personnel, and gilded subcommittees that are often non-operational[15]. In Nigeria, for example, Fadare et. al. (2018) in their study to establish the presence and functionality of MTCs in the country found minimal presence of MTCs in many of the surveyed hospitals, often characterized by limited or non-functioning subcommittees, this alludes to many countries including Brazil, Jordan, Sierra-Leone among others [8,14,16].

In Uganda, just like in other LMICs, the low prevalence and suboptimal functioning of MTCs is a common phenomenon [15]. Inappropriate medicine use problems, burdened with antimicrobial resistance (AMR) leading to treatment failure, length of hospital stays, duration of treatment, cost of treatment, and drug toxicity and fatalities [17], characterizes many public health facilities (HF). The inability by the HFs to develop hospital formularies, polypharmacy, inadequate labelling of medicines in stores, overuse of antibiotics and low adherence to standard treatment guidelines are still being reported in many studies conducted about medicines use in HFs in Uganda [18–20].

The Uganda national medicines policy (NMP) 2015 spells out the intent to fight against inappropriate medicine use by the MoH as this would lead to the attainment of the highest standards of health for the population of Uganda, thereby allowing maximum therapeutic benefits from medicines through their scientifically sound and cost-effective use by the prescribers, dispensers, and the patients [21]. MoH came up with strategies to ensure appropriate medicines use including the creation of appropriate medicines use (AMU) programmes at national and in HFs, strengthening of the national pharmacovigilance system, promoting consumer awareness, and enforcing use of prescription forms for dispensing of medicines in HFs [21]. The central body to implement such strategies within the HF is the MTC; however, very few public HFs in Uganda have functional MTCs. In many HFs, MTCs

are either partially functional, composed of members who either lack the zeal and prowess to perform the assigned MTC roles or are inaptly appointed, and in some HFs these committees are completely dormant. Generally, the absence of MTC and its subcommittees compromises the quality of medicines prescribing, access and management of medicines within the HF since such committees provide a forum used by health care providers to facilitate knowledge sharing, professional development as well as encouraging mentorship through continuous medicines education (CMEs) [15,22,23].

In the early 2000s, the MoH embarked on a process to establish MTCs in hospitals in Uganda, with the aim of addressing inappropriate medicine use. It was hoped that, with the support from policies such as the NMP, HFs would embrace this strategy and so become a norm for any HF to have a functional MTC, however, this is still farfetched [1]. Several policy documents, including the MTC manual (2018) have been prepared and disseminated by the MoH to provide technical guidance on the establishment and functionalisation of the MTCs. This is coupled with the MoH collaborating with partners in the health sector such as global fund (GF), infectious diseases institute (IDI), and management sciences for health (MSH) to provide both financial and technical support to establish or revitalise MTCs in HFs. Often, authors conducting research on the functionality of MTCs in HFs in Uganda largely focus on the alignment of MTC guidelines to existing structure and scope of the committees without necessarily attempting to unearth factors hindering optimal performance of MTCs. Furthermore, these studies are usually carried out on a small sample of HFs, lacking a national representativeness.

Addressing the issues of medicines use problems not only requires a properly functioning MTC, but also, support from HF management through adequate financing of the MTC to conduct its core functions, including undertaking targeted interventions such as carrying out medicines use surveys, and continuous medicines education. In 2023, national medical stores

(NMS), MoH conducted a study on the prevalence and functionality of MTCs in HFs and these found sub-optimal performance in addition to non-existence in some HFs. This study, therefore, used panel data strategy on a nationwide coverage of public health facilities to establish the trend in the functionality of MTCs in Uganda and its associated factors between the two interventional waves. In this study the current functioning of MTCs was established including the suboptimal performance of its subcommittees. In general, all HFs currently have MTC structures in place, attributed to interventions from key players in the health sector.

2.0. Material and Methods

This section explains the methodological techniques that were used in this study, highlighting the study approach and design, data collection and management techniques as well as the analytical framework.

2.1 Study Design

This was a longitudinal assessment study, following up on the same panels for which data were collected at baseline. The panel in this case was the health facilities. The study followed up on "wave one," known as baseline. Therefore, panel data analysis techniques were used to compare results between the two waves. This study was quantitative in nature and utilised quantitative data analysis methods. The panel data in this study contained data points for each HF (panel) observed at two points in time. This called for complex stochastic estimation model specifications as illustrated in the analytical framework.

2.2 Study Area and Target Population

This panel study was conducted in all public HFs including health center IVs (HC4), general hospitals (GH), regional referral hospitals (RRH), national referral institutes (NRI), and national referral hospitals (NRH). HFs under the Ministry of defense and veteran affairs (MoDVA) did not participate in this study. These were not part of the study design right from

the inception phase due to their operational uniqueness. The target participants included MTC chairpersons, MTC secretaries, MTC members, hospital directors, medical superintendents, HC4 In-charges and Dispensers. In most HFs, pharmacists double as MTC secretaries. One response was submitted from each HF.

2.3 Sampling Procedure

The study covered HC4s, GHs, RRHs, and NRs excluding those that belong to MoDVA. A 100% response rate was achieved. The difference in the total sample between the two waves is because in wave two the Government of Uganda downgraded 2 GHs to HC4 level, 1 HC4 was upgraded to GH level, and 1 HC4 was introduced into the HC4 level from HC3 level.

Table 1: Number of HFs Interviewed

Level of care	Wave One	Wave Two	% Achieved
NRI	1	1	100.0%
NRH	5	5	100.0%
RRH	17	17	100.0%
GH	49	48	100.0%
HC4	194	194	100.0%
GRAND TOTAL	266	265	100.0%

2.4 Data Collection Process

The study used a semistructured survey questionnaire to collect data from the study respondents. The semistructured questionnaire was developed by the research team for wave one and was adopted by the same research team for this follow-up study. The questionnaire was structured into three main themes including MTC structure, MTC operations, and medicines use stewardship. The MTC structure was premised on the MTC mandate, a nationally adopted framework in which MTCs in Uganda operate including the terms of reference (ToRs), appointment of MTC members, MTC composition, and subcommittees. The MTC operations section, on the other hand, looked at the day-to-day activities of the committee, including MTC meetings, planning and budgeting, development of policies and procedures, and development of a formulary list. Finally, the medicines use stewardship in this

study was scoped on problem identification and investigation as well as MTC interventions through routine and ad-hoc surveys. The face-to-face interview method was used, facilitated by Kobo tool kit, a web-based data collection engine.

2.5 Data Management and Analysis

The data collected were transferred from the kobo toolbox into STATA 18.5 (statacorp Texas, USA). The data cleaning process encompassed data editing and the variable transformation processes including defining dummy variables for categorical regressors. Univariate analysis encompassed generating descriptive summary statistics (means/median, percentages and frequencies). Bivariate data analysis techniques were utilized to establish the statistically significant relationship between any two variables of interest. Two chi-square tests were used to determine the significance level of association between any two variables analyzed at the bivariate level. The type of chi-square used was dictated by the structure of the two variables under consideration. For example, where both variables followed a multinomial distribution or nominal in nature, Pearson's chi-square test was used to test for independence. In addition, for variables that exhibited a natural ordering among the categories, a linear-by-linear chi-square test was used to test for a linear relationship, especially where the outcome variable was the variable of interest. The ordinal linear-by-linear trend test usually exhibits greater power compared to testing for independence when the association has a positive or negative trend.

2.6 Analytical Framework

Functional status was constructed on a four-point scale where 1=lack of MTC, 2=Yes, MTC available but not functional, 3=Yes, MTC available but partially functional, and 4=MTC, fully functional. The response variable "functionality" depicts a natural ranking, conforming to a natural ordering. This was therefore coded as consecutive integers starting at 1 through 4. Ordered outcome variables most times violate the assumption of linear regression models and often produce inefficient estimates providing incorrect conclusions, as these variables portray

nonlinear relationships [24,25]. Therefore, to determine the factors influencing the performance of MTCs in HFs, a nonlinear ordinal-logistic regression model (ORM) was used.

Secondly, this study was conducted as a follow-up assessment, following up on the same HF MTCs that were covered in wave one. This offered an opportunity to explore the course of change in the functionality among the panels between the two waves. Therefore, there are specific statistical techniques designed to handle panel data; subject specific (SS) and population average (PA) models. In this study the SS model was used as this would properly allow exploring statistical dependence commonly known as "*autocorrelation*" among the repeated responses within subjects that must be accounted for using an appropriate statistical model rather than assuming average dependence among the repeated responses, which is often the case with PA models [26–28]. Second, the SS model allowed use of the random effects (RE) model, thus facilitating the exploration of time invariant predictors.

2.6.1. Estimation Process

The estimation process involved two stages. Bivariate analysis was used in the first stage, followed by model estimation at multivariate level.

a. Bivariate analysis

Two types of relationships were tested namely, test for linear relationship/trend and test for independence.

Test for linear relationship

Ordinal variables tend to exhibit a trend association between them [29]. Since the dependent variable was ordinal in nature and there were some ordinal predictor variables, the following linear-by-linear chi-square was used to test for a linear trend between variables that exhibited ordinal nature.

$$\mathbf{M^2 = (\boldsymbol{n} - 1)\boldsymbol{r}^2}$$

Where; $r = \frac{\sum_{i,j}(u_i - \bar{u})(v_j - \bar{v})\, p_{ij}}{\sqrt{\left[\sum_i (u_i - \bar{u})^2 p_{i+}\right]\left[\sum_j (v_j - \bar{v})^2 p_{j+}\right]}}$,

$u_1 < u_2 < u_3 < \cdots < u_i$ denote scores for rows

$v_1 < v_2 < v_3 < \cdots < v_j$ denote scores for columns

$\bar{u} = \sum_i u_i\, p_{i+}$ denote the sample mean of the row scores

$\bar{v} = \sum_j v_j\, p_{j+}$ denote the sample mean of the column scores

$\sum_{i,j}(u_i - \bar{u})(v_j - \bar{v})\, p_{ij}$ denote the sample *covariance* of *X* and *Y*

The scores have the same ordering as the category levels.

The above linear-linear chi-square test is valid only if the following assumptions hold:

a) Both the dependent and independent variables are linear in nature

b) The dependent variable is ordinal, and the independent variable may be nominal but with no more than two categories.

Test for Independence

Pearson's chi-square test was used to determine the association between the explanatory variables and the functionality status variable. Pearson's chi-square formula below was used.

$$\boldsymbol{x^2}_{(\boldsymbol{r-1})(\boldsymbol{c-1})} = \sum_{\boldsymbol{i=1}}^{\boldsymbol{r}} \sum_{\boldsymbol{j=1}}^{\boldsymbol{c}} \frac{(\boldsymbol{O_{ij}} - (\boldsymbol{E_{ij}}))^2}{(\boldsymbol{E_{ij}})}$$

O_{ij}=Total observed frequency for category j, in row i

E_{ij}=Total expected frequency for category j, in row i

Non-Parametric Analysis of Equality of Group Medians

Wilcoxon rank-sum test, a distribution free test, was used to test for the equality of group medians using ranks between the median number of MTC members by level of care. The statistical significance was established using 5% level of significance.

b. *Model Specification and Estimation*

The ordinal logistic regression model (ORM) was used at the multivariate level to estimate the effect of the predictor variables on the functionality status of HFs. Panel data techniques in STATA were used to fit a random effects model with ORM as the link function and in this case, larger values on the functionality scale were assumed to correspond to higher outcomes. The conditional distribution of the dependent variable given the random effects is assumed to be multinomial with success probability determined by the logistic cumulative distribution function[30]. A random effects model was used to estimate the subject specific effects.

$$\Pr(y_{it} > k|\kappa, X_{it}, \nu_i) = H(X_{it}\beta + \nu_i \quad (1)$$

for i=1,…,n panels, where t=1,…,n_i, ν_i are independent and identically distributed $N(0, \sigma_\nu^2)$, and κ is a set of cutpoints κ_1, κ_2, and $, \kappa_3$ and $H(.)$is the logistic cumulative distribution function.

Therefore, the probability of observing any functional status for a given HF at any given wave is:

$$p_{itk} \equiv \Pr(y_{it} = k|\, \kappa, X_{it}, \nu_i) = \Pr(k_{k-1} < X_{it}\beta + \nu_i + \epsilon_{it} \leq k_k) \quad (2)$$

$$p_{itk} \equiv \Pr(y_{it} = k|\, \kappa, X_{it}, \nu_i) = H(k_k - X_{it}\beta - \nu_i) - H(k_{k-1} - X_{it}\beta - \nu_i \quad (3)$$

$$p_{itk} \equiv \Pr(y_{it} = k|\, \kappa, X_{it}, \nu_i) = \frac{1}{1+\exp(-k_k + X_{it}\beta + \nu_i)} - \frac{1}{1+\exp(-k_{k-1} + X_{it}\beta + \nu_i)} \quad (4)$$

Where k_0 is taken as $-\infty$ and k_K is taken as $+\infty$. Here X_{it} does not contain a constant term as this is absorbed into the cut points[30]. In this study, the number of HFs, $i = 265$; time, $t = 2$; and categories of functionality, $k = 4$.

2.7 Ethics approval and consent to participate

The study was conducted in accordance with the principles of the Helsinki Declaration. All participants provided written informed consent, and the study was approved by the research and ethics committee (REC) of Makerere University School of Health Sciences with reference number MAKSHSREC-2024-768 The study was further approved by the Uganda National Council for Science and Technology, under approval number HS5391ES. The process of recruiting study participants started on Monday 10th February 2025 and continued through to Friday 21st February 2025.

At all levels, confidentiality was maintained intentionally to protect respondents who volunteered to provide information pertinent to the study. The interviewers introduced the study to the participants, explaining the purpose of the study and the intended benefits. The participants were given five minutes to clearly understand the study and consent to participate. The interview process began only after written informed consent had been given by the participant.

3.0.Results

This chapter presents findings from wave two of the medicines and therapeutic committees in public HFs. This was a follow-up study providing the current functionality status compared to the baseline assessment, one year apart between the two waves. The results are presented in 6 subsections. Subsection 3.1 presents the primary demographics of the study population in the form of background characteristics pertaining to the primary units of analysis and the respondents. Subsection 3.2 describes the outcome variable in functionality and its limiting challenges. Subsections 3.3, 3.4 & 3.5 detail aspects of the MTC structure, MTC operations

and medicines use stewardship. In this subsection, we also tackle the bivariate analysis of association using appropriate statistical techniques for testing either independence or linear relationships. Finally, subsection 3.6 provides advanced inferential analysis, illustrating the model estimation framework that exhibits the net effect and direction of factors affecting the functionality of MTCs in HFs. The ordered logistics model was used, utilising subject specific techniques, under a random effects framework.

3.1. Background Characteristics

The study interviewed a total of 265 MTCs comprising 194 HC4s, 48 GHs, 17 RRHs, and 6 NRs. Overall, teaching HFs accounted for 14.3% (38/265) of the total sample while 85.7% (227/265) were non-teaching HFs. In most HFs, the MTC chairpersons are usually physicians, especially in RRHs and NRs, while pharmacists serve as the MTC secretary; this study mostly targeted either the MTC chairperson or the MTC secretary as the primary. Table 2 below shows that the highest percentage of respondents were MTC chairpersons (19.2%, 51/265) followed by assistant inventory management officers (18.1%, 48/265), dispensers (17.7%, 47/265), while pharmacists accounted for 17% (45/265). Furthermore, the highest proportion of respondents (35.5%,94/265) had served for over six years in their current positions. Similarly, 35.1% (93/265) had served for about three to five years.

Table 2: Summary of Background Characteristics (n=265)

	**Nature of HF**					
	**Non-Teaching HF**		**Teaching HF**		**Total**	
Level of Care	*Freq.*	*%*	*Freq.*	*%*	*Freq.*	*%*
HC4	188	96.9	6	3.1	194	100
GH	36	75	12	25	48	100
RRH	3	17.6	14	82.4	17	100
NR			6	100	6	100
Total	227	85.7	38	14.3	265	100
	**Interviewee position in the HF**					

Title	HC4 Freq.	GH Freq.	RRH Freq.	NR Freq.	Total Freq.	%
MTC Chairperson	44	6		1	51	19.2%
AIMO/IMO	43	5			48	18.1%
Dispenser	39	8			47	17.7%
Pharmacist	2	22	16	5	45	17.0%
MTC Member	30	4	1		35	13.2%
Director/Superintendent	30	3			33	12.5%
Other	6				6	2.3%
Total	194	48	17	6	265	100.0%

Years Served in the HF

Years	HC4 Freq.	GH Freq.	RRH Freq.	NR Freq.	Total Freq.	%
6 years and above	58	25	8	3	94	35.5%
3 to 5 years	66	16	8	3	93	35.1%
1 to 2 years	42	7			49	18.5%
Less than 1 year	28		1		29	10.9%
Total	194	48	17	6	265	100.0%

3.2. Functionality of MTCs

MTCs are oversight bodies in HFs constituted to steward inappropriate medicines use practices in the HF. For purposes of this report, a fully functional MTC was defined as one such committee that held regular meetings, maintained documentation of operations, and performed critical roles such as evaluating medicine use and logistical functions and or developing Institutional EMHS lists. A partially functional MTC was defined as one that regularly held meetings and performed some of the tasks inscribed in the WHO or MoH MTC guidelines. Finally, a non-functional MTC was defined as an MTC that has established structures in place but does not carry out MTC operations. Figure 1 below illustrates the distribution of the functionality status of MTCs in public HFs in relation to the MTC intervention by national medical stores, MoH, and development partners such as global fund (GF). The percentage of

HFs with a functional MTC increased significantly from 8.3% (22/264) in wave one to 20.8% (55/265) in wave two (p-value<0.05). The findings revealed a sharp fall in the proportion of HFs with no MTC in place from 72% (190/265) in wave one to only 7.6% (20/265) in wave two, demonstrating the impact of the intervention, particularly around establishing MTC structures. The biggest improvement in having a fully functional MTC between the two waves was observed in GHs (wave one=12.8%, wave two=56.7%). RRHs (wave one=52.9%, wave two=76.5%). As in the baseline, RRHs still reported the highest percentage of HFs with a fully functional MTC (13/17).

Figure 1: Functionality Status of MTCs in HFs

The results demonstrate a positive impact from the intervention, especially at HC4 level where the MTC structure is now in place unlike in the baseline assessment. Noticeable performance was also demonstrated at the GH level, where nearly all of them have changed to at least having an MTC in place.

3.2.1. *Testing for the distribution of functionality across levels of care*

The study further explored the extent of the linear relationship between the functional status of MTCs and the HF level of care. Functional status was the outcome variable in this study, while level of care was one of the predictors. Both variables exhibited a natural ordering. Whereas the Pearson chi-square is the most popular test for independence, it is however less superior in testing for linear relationships. Therefore, a linear-by-linear chi-square test was preferred. This was used to establish whether a linear trend existed between the two variables. The linear trend significance test was further applied on the wave number. Furthermore, a significantly positive relationship was established between the two waves and the functional status (p-value=0.00<0.05), demonstrating improved performance overtime.

Table 3Table 3 below shows that the mean score on the functional real line at the HC4 level of care in wave two was 2.27 (SD=0.72) compared to 1.14 (SD=0.55) in wave one. The mean

score at the GH level in wave two was 3.54 (SD=0.54) compared to 2.53 (SD=0.97) in wave one. The mean score at the RRH level in wave two was 3.76 (SD=0.44) compared to 3.47 (SD=0.62) in wave one. The mean score at the NR level remained the same in both waves (mean=3.67, SD=0.52). The functional status score increased with an increase in the level of care, exhibiting a statistically significant positive linear trend (p-value=0.00<0.05). This means that greater impact is currently realised in HFs that offer more specialised services with more specialist cadres who have a high-level understanding of the role of MTCs in stewarding appropriate medicines use and management. Furthermore, a significantly positive relationship was established between the two waves and the functional status (p-value=0.00<0.05), demonstrating improved performance overtime.

Table 3: Testing for the distribution of functionality across levels of care

**Distribution of functionality across levels of care (wave two)**										
**MTC functionality**	HC4		GH		RRH		NR		Total	
	Freq	%	Freq	%	Freq	%	Freq	%	Freq	%
No	20	10.3	0		0		0		20	7.5
Yes, but not functional	112	57.7	1	2.1	0		0		113	42.6
Yes, partially functional	51	26.3	20	41.7	4	23.5	2	33.3	77	29.1
Yes, fully functional	11	5.7	27	56.2	13	76.5	4	66.7	55	20.8
**Total**	**194**	**100**	**48**	**100**	**17**	**100**	**6**	**100**	**265**	**100**
**Testing for a linear trend in the mean scores on functionality across levels of care in the two waves**										
	**HC4**		**GH**		**RRH**		**NR**		**Total**	
Wave one: Mean (SD)	1.14 (0.55)		2.53 (0.97)		3.47 (0.62)		3.67 (0.52)		1.6 (1.02)	
Wave two: Mean (SD)	2.27 (0.72)		3.54 (0.54)		3.76 (0.44)		3.67 (0.52)		2.63 (0.9)	
Linear by Linear Chi-square: by level of care									9.804 (0.0000*)	
Linear by Linear Chi-square: by wave									10.879 (0.0000*)	

Source: Primary data; *Significant at 5%

3.2.2. *MTC functionality transitional probabilities*

Table 4 below describes the likelihood with which HF MTCs moved from one category of functionality into another, below or above the category. The rows reflect numbers in wave one while the columns represent final destinations. For example, in wave one, 190 HFs lacked an MTC, however, in wave two, this number reduced significantly to 20 HFs. 55.3% (105/190) of the HFs that lacked an MTC at baseline had established MTCs in place, although they were not

functional, 25.3% (48/190) transitioned to partial functionality while 9% (17/190) transitioned to full functionality status. Table 4 below further shows that the probability of transitioning from having a partially functional MTC to a fully functional MTC considering wave one and wave two results was 52.5% higher compared to transitioning from having a nonfunctional MTC to a fully functional MTC (33.3%).

Table 4: MTC functionality transitional probabilities

MTC functionality	No		Yes, but not functional		Yes, partially functional		Yes, fully functional		**Wave one**	
	Freq.	%	Freq.	%	Freq.	%	Freq.	%	Freq.	%
No	20	10.5	105	55.3	48	25.3	17	9.0	**190**	100.0
Yes, but not functional	0	0.0	3	25.0	5	41.7	4	33.3	**12**	100.0
Yes, partially functional	0	0.0	4	10.0	15	37.5	21	52.5	**40**	100.0
Yes, fully functional	0	0.0	1	4.6	8	36.4	13	59.1	**22**	100.0
**Wave two**	**20**	**7.6**	**113**	**42.8**	**76**	**28.8**	**55**	**20.8**	**264**	**100.0**

3.2.3. Reasons for the lack of a functional MTC

The operational status of MTCs depends on a multitude of factors, some internal to the HF while others are global, presumably external to the HF. Table 5 below presents some of the challenges that maybe contributing to the suboptimal performance of MTCs in HFs. These are distributed both by wave and level of care. Statistical significance between waves was tested using Pearson's chi-square test at a 95% level of confidence. It was established that lack of adequate knowledge in MTC operations (wave one=89.4%, wave two=73.1%) and lack of financial support (wave one=64.8%, wave two=51.9%) are still prevalent in hampering MTC performance in the HFs. All the challenges saw a downward trend between the two waves and the differences in each of the challenges between the two waves were statistically significant (p-value=0.00<0.05) except the challenge of lack of commitment from MTC members (p-value=0.753>0.05). Overall, the challenges facing the operational status of MTCs in HFs are gradually waning. Table 5 below further shows that these challenges are more pronounced in HC4s, and GHs to a lesser extent, but almost near to no concentration in RRHs and NRs. The

zeal and focus to operationalize MTCs should be targeted to HC4s rather than any other level of care, especially leveraging the advantage that the MTC structure is in place for majority of the HC4s and existence of commitment from the MTC members.

Table 5: Distribution of challenges for lack of a functional MTC by wave and level of care

**Distribution of challenges by wave (%)**				
**Challenge**	**Wave one**	**Wave two**	**P-value**	**Conclusion**
Knowledge gap in MTC operations	89.4	73.1	***0.000****	*Statistically significant*
lack of financial support	64.8	51.9	***0.006****	*Statistically significant*
Lack of commitment from members	29.2	27.9	***0.753***	*Not statistically significant*
Lack of will and support from hospital administration	8.1	15.4	***0.016****	*Statistically significant*
Other specify	14.4	42.8	***0.000****	*Statistically significant*
**Overall p-value**			**0.000***	*Overall, statistically significant*

**Distribution of challenges by level of care (%)**								
	**HC4**		**GH**		**RRH**		**NR**	
	Wave one	Wave two	Wave one	Wave two	Wave one	Wave two	Wave one	Wave two
Knowledge gap in MTC operations	77.5	63.0	10.6	8.7	0.9	1.0	0.4	0.5
lack of financial support	49.2	43.3	11.9	6.7	3.0	1.0	0.9	1.0
Lack of commitment from members	18.6	20.7	8.5	5.3	2.1	1.4	0.0	0.5
Lack of will and support from hospital administration	3.0	11.5	4.2	3.4	0.4	0.5	0.4	0.0
Other specify	12.3	38.9	1.7	3.4	0.0	0.5	0.4	0.0

Source: Primary Data; *Significant at 5%

3.3. *MTC Structure*

The MTC is a multidisciplinary body composed of physicians, pharmacists, hospital administrators, laboratory technicians, nurses, medical records officers among others. In an organized MTC structure, these should be appointed officially with clear terms of reference. The MTC structure also encompasses the availability of policy and procedure documents and the presence of subcommittees. This section provides up-to-date information regarding the MTC structure comparing the two waves.

3.3.1. *Appointment of MTC members*

The study explored the extent to which HF staff are appointed to formally serve as MTC members. Figure 2 below shows a great improvement in the appointment of MTC members. Overall, 86% of MTCs had members officially appointed in wave two compared to only 35.7%

in wave one. All levels of care scored above 80% with RRHs having the highest percentage of officially appointed members (wave one=68.8%, wave two=94.1%) followed by GHs (wave one=26.5%, wave two=91.7%). HC4s recorded the biggest improvement between the two waves, moving from no MTCs with officially appointed members in wave one to 83.7% in wave two.

Figure 2: Appointment of MTC members (n=265)

3.3.2. *Level of member participation*

Figure 3 shows some of the improvements in the presence of active MTC members. The findings indicate that the number of HFs with all MTC members actively participating in MTC operations increased from 25.7% in wave one to 51.2% in wave two. This change benefited from the reduction in the number of members who transitioned from being partly active in wave one (wave one=62.9%, wave two=38.5%). Pearson's chi-square test was used to test for the significance of the changes between the two waves using 95% level of confidence. Generally, the changes between the two waves were statistically significant (p-value=0.001<0.05).

Figure 3: Level of member participation

3.3.3. *Average Number of MTC Members*

The MoH manual on MTCs recommends that the number of MTC members may range between 12 and 15. Additionally, members can be co-opted by the MTC or its sub-committees where necessary for specific matters, although these should not have voting powers [31]. Table 6 below provides the median number of MTC members for each level of care and the difference between the two waves. The median number of MTC members was highest at the RRH level in both waves and the gaps are so wide compared to other HF levels of care including NRs. Overall, the median number of participants was 13 (min=0, max=32) in wave one compared to 15 (min=5, max=30) in wave two. The median number in wave one and wave two conform to

the MOH recommended number. The study went further to statistically establish whether the median numbers significantly varied between the two waves. Wilcoxon rank-sum test, a distribution free test was used to test for the equality of group medians using ranks. Results in the table below revealed that the median number of MTC members between the two waves was significantly different from each other (p-value=0.0112<0.05). This means that the intervention to revitalize the MTCs after the baseline assessment significantly increased the motivation to join the MTCs, especially at HC4 and GH level.

Table 6: Median number of MTC members by level of care

Level of care	Wave One			Wave Two		
	**Median**	**Min**	**Max**	**Median**	**Min**	**Max**
HC4	9.5	0	30	14	5	22
GH	12	5	23	16	11	27
RRH	18	15	32	19	15	30
NR	12.5	10	32	15	13	22
Total	13	0	32	15	5	30
Wilcoxon rank-sum test					0.0112*	

Source: Primary data; Significant at 5%.

3.3.4. *Availability of Policies and Procedures.*

The operational activities of MTCs as prescribed in the MoH MTC manual are guided by policies and standard operating procedures. One of the challenges to effective MTC operations is the lack of clear instructions and guidelines[31]. Some of the guidelines are adopted from the national level such as the antimicrobial stewardship (AMS), pharmacovigilance (PV) guidelines, MTC manual, EMHS list and the national standard treatment guidelines (STGs) while others like the terms of reference (ToRs) maybe developed by the HF or adopted and customised. Figure 4 below shows the trend in the availability of policies and procedures in HFs between the two waves. The ToRs and MTC guidelines recorded the highest percentage change in their availability in HFs between the two waves (wave one=44.6%, wave two=88.2%) and (wave one=66.2%, wave two=82.5%) respectively, while PV (wave one=46.0%, wave two=27.8%) and AMS (wave one=24.3%, wave two=13.9%) guidelines

registered a significant decline. STG and EMHS list maintained a high presence between the two waves.

Figure 4: Percentage availability of policies and procedures.

The study further explored the statistical significance of the changes in the availability of policies and procedures between wave one and wave two. Pearson's chi-square test was used. Overall, the changes in the availability of policies and procedures between wave one and wave two were statistically significant (p-value=0.00<0.05). This means that as we move from the initial stages of the intervention into the operational phase, availing policy documents to HF MTCs to guide their operations is paramount. At the group level, Table 7 below shows that the relationship between the availability of EMHS list and the two waves was not significant (p-value>0.05) otherwise all other changes were significant.

Table 7: Availability of policies and procedures.

**Guidelines**	**Cases (Frequency)**		
	**Wave one**	**Wave two**	**Group p-value (unadjusted)**
MTC guidelines	49	202	0.003*
PV guidelines	34	68	0.003*
AMS guidelines	18	34	0.031*
STG guidelines	60	225	0.009*
EMHS list	62	206	0.974
ToRs	33	216	0.000*
Cases: Overall P-value (adjusted)	73	243	0.000*

Source Primary data; *significant at 5%

3.3.5. Availability of MTC subcommittees

Table 8 below illustrates the extent to which MTC subcommittees are available and their functional status in the HFs, disaggregated by wave number and level of care. In general, almost all subcommittees observed a reduction under full functionality. This downward trend is attributed to the reduction in the availability of functional subcommittees at the HC4 level. The supply chain subcommittee is the most prevalent, although its full functionality status declined from 37.0% in wave one to 25.3% in wave two. Besides the research subcommittee, all other subcommittees are in place regardless of operational status, unlike in wave one where lack of subcommittees in place was more prevalent. The highest presence and improvement were

observed at the RRH level, just like in wave one. The reactivation of the operational status of the subcommittees requires strengthening at all levels of care. The subcommittees are the operational arm of the main MTC, their activity outputs are inputs into the main MTC work. Therefore, their absence adversely affects the main MTC activities.

Table 8: Availability of MTC subcommittees (wave two, n=265)

	**Availability of PV subcommittee**									
	HC4		GH		RRH		NR		Total	
Functionality status	Wave one	Wave two	Wave one	Wave two	Wave one	Wave two	Wave one	Wave two	Wave one	Wave two
Very Functional	21.4	1.2	16.2	35.4	29.4	58.8	16.7	16.7	20.3	12.3
Partially functional	14.3	16.8	24.3	41.7	41.2	23.5	50.0	50.0	28.4	23.0
Available but not functional	14.3	74.0	5.4	18.8	29.4	17.7	33.3	16.7	14.9	57.8
Not in place	50.0	8.1	54.1	4.2	0.0	0.0	0.0	16.7	36.5	7.0
Total	100.0	100.0	100.0	100.0	100.0	100.0	100.0	100.0	100.0	100.0
	**Availability of AMS subcommittee**									
	HC4		GH		RRH		NR		Total	
Functionality status	Wave one	Wave two	Wave one	Wave two	Wave one	Wave two	Wave one	Wave two	Wave one	Wave two
Very Functional	7.1	2.3	2.9	20.8	17.7	52.9	33.3	33.3	9.7	10.3
Partially functional	21.4	11.0	8.6	45.8	58.8	35.3	16.7	50.0	23.6	20.5
Available but not functional	21.4	78.0	8.6	29.2	23.5	11.8	50.0	16.7	18.1	62.3
Not in place	50.0	8.7	80.0	4.2	0.0	0.0	0.0	0.0	48.6	7.0
Total	100.0	100.0	100.0	100.0	100.0	100.0	100.0	100.0	100.0	100.0
	**Availability of supply chain subcommittee**									
	HC4		GH		RRH		NR		Total	
Functionality status	Wave one	Wave two	Wave one	Wave two	Wave one	Wave two	Wave one	Wave two	Wave one	Wave two
Very Functional	21.4	8.6	37.8	66.7	56.3	70.6	16.7	50.0	37.0	25.3
Partially functional	35.7	19.0	21.6	16.7	18.8	17.7	50.0	33.3	26.0	18.8
Available but not functional	21.4	64.9	8.1	10.4	18.8	11.8	33.3	16.7	15.1	49.4
Not in place	21.4	7.5	32.4	6.3	6.3	0.0	0.0	0.0	21.9	6.5
Total	100.0	100.0	100.0	100.0	100.0	100.0	100.0	100.0	100.0	100.0
	**Availability of Research subcommittee**									
	HC4		GH		RRH		NR		Total	
Functionality status	Wave one	Wave two	Wave one	Wave two	Wave one	Wave two	Wave one	Wave two	Wave one	Wave two
Very Functional	0.0	0.0	2.8	0.0	6.3	12.5	16.7	20.0	4.3	1.3
Partially functional	0.0	0.0	5.6	2.2	25.0	12.5	33.3	20.0	11.4	1.7
Available but not functional	16.7	14.6	5.6	6.7	12.5	31.3	0.0	0.0	8.6	13.9

Not in place	83.3	85.4	86.1	91.1	56.3	43.8	50.0	60.0	75.7	83.1
Total	100.0	100.0	100.0	100.0	100.0	100.0	100.0	100.0	100.0	100.0

Source: Primary data

3.4. MTC Operations

The MoH recommends the establishment of vibrant MTCs in each HF with the intent to spearhead rational medicines use in the HFs. Routine MTC activities include planning and budgeting, conducting meetings, formulating policies, and carrying out interventions. The subsections below present the work of MTCs in public HFs regarding the MTC operations.

3.4.1. *Planning and Budgeting*

The MoH MTC guidelines 2018 prescribe that a functional and effective MTC should be able to plan and budget for its activities. The MTC work plan should then be incorporated into the HF workplan. It is the responsibility of the HF to facilitate the MTC activities. Table 9 below shows an upward trend in the percentage of MTCs with a workplan and budget included in the HF workplan and budget for the MTC work (wave one=16.2%, wave two=27.1%). The availability of a workplan and a budget for the MTC work is significantly associated with an increase in wave number (p-value=0.000<0.05). Any further interventions as time goes by are likely to positively influence the formulation of MTC workplans and budgeting to smoothly run the MTC work.

The proportion of MTCs with a workplan and a budget in place, although not included in the HF workplan, was 11.5%. This means that for such MTCs, their support, whether in kind or financial, is borne by other parties external to the HF. Of course, development partners like IDI and the global fund are supporting some MTC activities.

GHs (wave one=8.1%, wave two=43.8%), RRHs (wave one=23.5%, wave two=41.2%), and NRs (wave one=16.7%, wave two=33.3%) had the highest percentage of MTCs whose workplan is incorporated into the HF workplan compared to their counterparts at HF level (wave one=28.6%, wave two=20.8%) that recorded a shortfall of nearly 8 percentage points.

More support is needed at the HC4 level to redress and avert their current performance. This may require not only financial support but also capacity building through mentorship, coaching, and benchmarking.

Table 9: Availability of budget and workplan (n; wave one=74, wave two, n=244)

	MTC has a work plan and a budget		MTC has a work plan and a budget but it is not included in the HF work plan and budget		MTC has a work plan but without a budget		No	
Level of Care	Wave one	Wave two	Wave one	Wave two	Wave one	Wave two	Wave one	Wave two
HC4	28.6	20.8		5.8	7.1	8.7	64.3	64.7
GH	8.1	43.8		25.0	24.3	8.3	67.6	22.9
RRH	23.5	41.2		17.7	47.1	23.5	29.4	17.7
NR	16.7	33.3		50.0	50.0	0.0	33.3	16.7
Total	16.2	27.1		11.5	28.4	9.4	55.4	52.1
Waves: Chi-square (p-value)	26.07 (0.0000*)							

Source: Primary data; *significant at 5%

3.4.2. *Funding for MTC Activities*

Additionally, the study explored the funding sources for MTC activities. Figure 5 below shows an upward trend in the funding for the MTC activities by the HFs (wave one=54.6%, wave two=73.1%). This demonstrates increased commitment to MTC activities by the HF administration but also highlights indications for future sustainability of the MTC program. Other funders include NMS (wave two=61.3%), which started its support shortly after the inception of the revitalization program activities.

Kawempe NRH indicated that its MTC was being supported by the Commonwealth Pharmacists Association, the Tropical Health and Education Trust, the Fleming Fund and Cambridge Global Health Partnerships. Butabika NRH and Jinja RRH indicated receipt of support from the national drug authority (NDA).

Figure 5: Sources of funding for MTC activities

3.4.3. External Support for MTC Activities

Table 10 below shows the kind of support MTCs received between wave one and wave two. In wave one, the highest concentration of support was observed in RRHs, where, for example, all external financing to MTCs went to RRHs. Wave two depicted a relatively better approach, where the highest concentration of support in terms of financing, capacity building or both largely shifted to HC4s and GHs. This clearly explains the improved performance in the functionality of MTCs we saw in HC4s and GHs in section 3.2 above.

The analysis of the association between external support and level of care was presented on two fronts. First, in wave one, the results in the table below indicate that there was no significant relationship between external support and level of care (p-value=0.20>0.05). This is because the distribution of external support was the same in all other levels of care except at the RRH level. Secondly, in wave two, the interventions by implementing partners laid a levelled ground especially at GHs and HC4s. The distribution of support moved downwards with lower-level HF MTCs more likely to get more support than higher-level HF MTCs and this relationship was significant (p-value=0.0000<0.05). While this support has significantly achieved greater heights in establishing MTC structures in HC4s and GHs, the focus should now shift to operationalising them. Implementing partners supporting the MTC activities include IDI; GF; and medicines, technologies, and pharmaceutical services (MTaPS); NMS; Makerere University school of public health; Medical team international; and Baylor Uganda.

Table 10: Support for MTC activities disaggregated by wave and Level of care

**Wave One: MTC support by level of care (n=37)**						
	HC4	GH	RRH	NR	Total	P-value
Capacity building	12.0	32.0	44.0	12.0	100.0	0.262
Financial support	0.0	0.0	100.0	0.0	100.0	0.110
Capacity building and financial support	33.3	16.7	33.3	16.7	100.0	0.634
Other	44.4	44.4	0.0	11.1	100.0	0.036*
Total	20.9	30.2	37.2	11.6	100.0	

Wave One: Chi-square (p-value)					22.75 (0.200**)	
	**Wave Two: MTC support by level of care (n=226)**					
	HC4	GH	RRH	NR	Total	P-value
Capacity building	79.0	15.6	4.2	1.2	100.0	0.000*
Financial support	20.6	58.8	14.7	5.9	100.0	0.000*
Capacity building and financial support	42.7	37.3	16.0	4.0	100.0	0.000*
Other	42.9	42.9	14.3	0.0	100.0	0.348
Total	61.5	27.2	8.8	2.5	100.0	
Wave One: Chi-square (p-value)					118.2 (0.000**)	

Source: Primary data; *significant at 5%; **Overall p-values are adjusted p-values

3.4.4. *MTC meetings*

The MoH MTC manual (2018) stipulates that an effective MTC should regularly hold meetings entailing preparing and communicating the meetings, managing and controlling, and documentation of the meeting minutes. The manual recommends holding meetings bimonthly.

Figure 6 below illustrates the frequency of MTC meetings in HFs. NRs and RRHs had a more structured and appealing frequency of holding MTC meetings in both waves.

Among the NRs, in wave two, 66.7% (4/6) conducted MTC meetings quarterly while Butabika NRH and Mulago NRH held MTC meetings bimonthly and monthly respectively. Among the RRHs, the proportion of MTC meetings held bimonthly as stipulated in the MTC manual increased from 50% (8/16) to 58.8% (10/17) in wave two while those holding meetings quarterly increased from 12.5% (2/16) in wave one to 35.3% (6/17) in wave two. Improvement was observed in GHs where most MTCs held meetings (50%, 24/48) quarterly and 33.3% (16/48) held MTC meetings bimonthly in wave two compared to 22.9% (8/35) and 5.7% (2/35) in wave one respectively. Re-enforcement is needed at the HC4 level. Additionally, the percentage of MTCs documenting their meeting minutes reduced from 77.0% (57/74) in wave one to 61.5% (150/244) in wave two.

Figure 6: Frequency of MTC meetings in HFs

The study further explored the significance of the relationship between the frequency of MTC meetings and the level of care. The frequency was numbered on a linear scale as 1= when need

arises, 2=rarely, 3=biannual, 4=quarterly, 5=bimonthly, and 6=monthly. A linear-by-linear chi-square test, a measure of trend among ordered variables, was used. The findings in Table 11 demonstrate a statistically significant association between the frequency of MTC meetings and the HF level of care (p-value=0.024<0.05). This means that currently, MTCs in NRs, RRHs, and GHs in that order, are more likely to hold MTC meetings regularly compared to their counterparts at the HC4 level.

Table 11: Test for linear trend between level of care and frequency of MTC meetings

Test for linear trend between level of care and frequency of MTC meetings				
Level of care	Wave one		Wave two	
	Freq	Mean Score (SD)	Freq	Mean Score (SD)
HC4	13	3.77 (1.54)	170	3.92 (1.3)
GH	35	2.77 (1.59)	48	4.21 (1.11)
RRH	16	4.63 (1.15)	17	4.71 (0.59)
NR	6	4.67 (0.82)	6	4.5 (0.84)
Total	70	3.54 (1.64)	241	4.05 (1.24)
Linear by Linear Z-score (P-value)			2.257 (0.0240*)	

Source: Primary data; *significant at 5%

3.4.5. *Development of Policies and Procedures within the HF*

The study collected data on the development of policies and procedures by the MTCs. MoH strongly recommends that HFs should either adopt or develop policies for the better management of EMHS within the HFs. Figure 7 below shows a general decline in the extent to which MTCs developed policies and procedures between the two waves. The percentage of HFs that had developed policies regarding prescription, dispensing, and administration of medicines reduced from 65.2% in wave one to 47.2% in wave two. If HFs are to tackle irrational use of EMHS, double efforts are needed to provide guiding documents to the health workforce within the HFs.

Figure 7: Development of policies and procedures within the HF

3.4.6. Planning and Stock Management Practices

Table 12 below shows significant improvement in the use of MTCs in planning and inventory management practices in the HFs. Across all the indicators studied, RRHs registered the highest percentage score closely followed by NRs between the two waves, although drastic positive changes were observed at GH level. In ensuring that the HF establish their EMHS needs, which eventually guides the development of the HF procurement plan, RRHs (wave one=75.0%, wave two=94.1%) had the highest number of MTCs that regularly get involved in the needs analysis activity, followed by GHs (wave one=27.0%, wave two=74.5%), while HC4 came last (wave one=42.9%, wave two=16.7%). In the procurement planning exercise, all RRH MTCs got involved per the wave two findings compared to 88.2% in wave one. NRs maintained the same percentage (83.3%) between the two waves while GHs registered a tremendous upward trend from 32.5% in wave one to 85.4% in wave two.

Table 12: Planning and stock management

**MTC was involved in the selection/needs analysis of EMHS list (n; wave one=73, wave two=244)**						
Level of care	No		Yes, regularly (yearly)		Yes, sometimes	
	Wave one	Wave two	Wave one	Wave two	Wave one	Wave two
HC4	50.0	77.6	42.9	16.7	7.1	5.8
GH	29.7	10.6	27.0	74.5	43.2	14.9
RRH		5.9	75.0	94.1	25.0	
NR		16.7	66.7	66.7	33.3	16.7
Total	24.7	58.2	43.8	34.4	31.5	7.4
**MTC was involved in planning for EMHS during procurement planning and quantification (n; wave one=74, wave two=245)**						
Level of care	No		Yes, regularly (yearly)		Yes, sometimes	
	Wave one	Wave two	Wave one	Wave two	Wave one	Wave two
HC4	35.7	61.5	50.0	27.0	14.3	11.5
GH	29.7	2.1	32.4	85.4	37.8	12.5
RRH	0.0	0.0	88.2	100.0	11.8	0.0
NR	16.7	16.7	83.3	83.3	0.0	0.0
Total	23.0	44.5	52.7	44.9	24.3	10.6
**MTC was involved in reviewing orders (n; wave one= 74, wave two=243)**						
Level of care	No		Yes, regularly (yearly)		Yes, sometimes	
	Wave one	Wave two	Wave one	Wave two	Wave one	Wave two
HC4	50.0	77.0	28.6	11.5	21.4	11.5
GH	46.0	39.1	18.9	45.7	35.1	15.2

RRH	35.3	17.7	29.4	58.8	35.3	23.5
NR	16.7	33.3	33.3	66.7	50.0	0.0
Total	41.9	64.6	24.3	22.6	33.8	12.8
**MTC was involved in oversight of EMHS use (n; wave on=0, wave two=243)**						
Level of care	No		Yes, regularly (yearly)		Yes, sometimes	
	Wave one	Wave two	Wave one	Wave two	Wave one	Wave two
HC4		73.5		16.5		10.0
GH		29.8		57.5		12.8
RRH		6.3		75.0		18.8
NR		16.7		66.7		16.7
Total		59.0		29.7		11.3
**MTC produces regular reports on stock availability and expiries of EMHS (n; wave one=74, wave two=242)**						
Level of care	No		Yes, regularly (yearly)		Yes, sometimes	
	Wave one	Wave two	Wave one	Wave two	Wave one	Wave two
HC4	42.9	75.4	35.7	11.7	21.4	12.9
GH	37.8	14.6	16.2	54.2	46.0	31.3
RRH	35.3	17.7	17.7	58.8	47.1	23.5
NR	16.7	16.7	33.3	66.7	50.0	16.7
Total	36.5	57.9	21.6	24.8	41.9	17.4

Source: Primary data

3.4.7. Development of an Institutional Medicines List

As one of the interventions, the HF MTC is responsible for producing an institutional EMHS list, derived from the national EMHS list, which serves as a guide for the indication of EMHS pertaining to conditions local to the surrounding environment. The findings in Table 13 revealed low availability of the institutional EMHS list in HFs. Overall, only 4.1% of HF MTCs had developed and updated their institutional EMHS list, a decline from 8.1% that was observed in wave one. Furthermore, the percentage of MTCs with an institutional EMHS list but not updated reduced from 13.9% in wave one to only 5.8% in wave two. Overall, by the time wave two was conducted, 90% of the surveyed HF MTCs lacked an institutional EMHS list. There was a statistically significant association between the development of an institutional EMHS list and level of care. As observed, the probability of finding an institutional EMHS list in RRHs and NRs is much higher compared to GHs and HC4s. While the situation is much worse at HC4 and GH levels, all levels of care require immediate assistance.

Table 13: Development of an Institutional Medicines list (n; wave one=72, wave two=242)

Level of care	No		Yes, regularly updated		Yes, but not updated	
	Wave one	Wave two	Wave one	Wave two	Wave one	Wave two
HC4	100.0	95.9	0.0	2.3	0.0	1.8
GH	80.0	81.3	2.9	4.2	17.1	14.6
RRH	64.7	70.6	17.7	11.8	17.7	17.7
NR	50.0	50.0	33.3	33.3	16.7	16.7
Total	77.8	90.1	8.3	4.1	13.9	5.8
Wave: Chi-square (p-value)					55.4273 (0.000*)	

Source: Primary data; *Significant at 5%

3.4.8. Development of an HF-based antibiogram

The antibiogram is an essential resource for institutions to track changes in antimicrobial resistance and to guide empirical antimicrobial therapy, commonly utilized to monitor recent antimicrobial susceptibility patterns to guide empirical antimicrobial therapy selection [32]. The findings in Table 14 below shows that only 2.1% of HF MTC had developed an antibiogram in wave two compared to 2.7% in wave one. None of the MTCs in GHs or HC4s had developed an updated antibiogram. A relatively better picture was observed in NRs (wave one=16.7%, wave two=33.3%). There was a statistically significant association between the development of an antibiogram and level of care (p-value=0.000<0.05). The probability of finding an antibiogram in NRs is much higher compared to RRHs, GHs, and HC4s.

Table 14: Availability of HF-based antibiogram (n; wave one=74, wave two=242)

Level of care	No		Yes, regularly updated		Yes, but not updated	
	Wave one	Wave two	Wave one	Wave two	Wave one	Wave two
HC4	100.0	97.7	0.0	0.0	0.0	2.3
GH	97.3	97.9	0.0	0.0	2.7	2.1
RRH	70.6	76.5	5.9	17.7	23.5	5.9
NR	66.7	66.7	16.7	33.3	16.7	0.0
Total	89.2	95.5	2.7	2.1	8.1	2.5
Wave: Chi-square (p-value)					64.0547 (0.0000*)	

Source: Primary data; *Significant at 5%

3.5. Medicines Use Stewardship

Medicines use stewardship is a term used to describe the coordinated strategies and interventions used to ensure medicines use, usually within a specific therapeutic area. The intention is usually to minimise variation in use but also to ensure improved patient outcome

and safety [33]. Improving medicines use stewardship is a continuous process with activities such as surveys and interventions, intended to improve prescribing and medication management at individual and population level to ensure consistent and appropriate care [33]. One example that is currently common in Uganda and being popularized by the MoH is antimicrobial stewardship.

3.5.1. *Conducting Surveys on Medicines Use*

Table below shows the extent to which HFs conducted surveys on medicines use, intended to combat irrational medicines use as well as to improve medicines management. The term regular was used to mean those MTCs that conduct any of these surveys at least once a year and the analysis below is more centric to regular performance.

Overuse of injectable medicines in OPD: The results below reveal a declining trend in conducting of surveys on overuse of injectable medicines in OPD between the two waves. The percentage of MTCs regularly conducting surveys on overuse of injectable medicines in OPD reduced from 16.2% in wave one to only 7.8% in wave two. The downward trend was accelerated by HC4s (wave one=7.1%, wave two=4.1%) and RRHs (wave one=41.2%, wave two=23.5%). NRs improved from 0.0% in wave one to 33.3% in wave two.

Recording of medicines administered in wards: The findings in the table below indicate a downward trend in conducting surveys on the recording of administered medicines in the wards. In wave one, the proportion of MTCs that were conducting surveys on the recording of medicines administered regularly in the wards was 23.3% compared to only 10.7% that was observed in wave two. The poor performance was powered by HC4s (wave one=15.4%, wave two=6.4%), GHs (wave one=21.6%, wave two=14.6%), and RRHs (wave one=41.2%, wave two=29.4%). The percentage of MTCs conducting this survey regularly at the NR level increased from 0.0% in wave one to 50% in wave two.

Overstock and understock of certain items: The study found that only 14.3% of the HF MTCs conducted surveys on the overstock and understock of EMHS in the HF. This is a decline from 47.3% in wave one.

Generally, whereas all levels of care recorded a downward trend in at least two of the three surveys under review, the performance of NRs was relatively eminent across all the three surveys. Finally, the Pearson's chi-square test was used to estimate the relationship between level of care and the score on the surveys being conducted. The findings below show that across all three surveys, a statistically significant positive association between the two variables exists (p-value<0.05). This means that the intensity of the MTC intervention is more likely to impact greater performance in conducting surveys at NRs moving downwards. This could be attributed to the availability of specialists in higher level HFs, the huge number of MTC members in such HFs, and a high level of commitment. Also, the MTCs in NRs and RRHs are more functional compared to GHs and HC4s, therefore more likely to be involved in the wider scope of MTC activities.

Table 15: Surveys on Medicines Use

Overuse of injectable medicines and antibiotics in OPD (n; wave one=74, wave two=244)						
Level of Care	Irregular		No		Yes, regular (annually)	
	Wave one	Wave two	Wave one	Wave two	Wave one	Wave two
HC4	28.6	5.2	64.3	90.8	7.1	4.1
GH	27.0	27.1	62.2	60.4	10.8	12.5
RRH	41.2	35.3	17.7	41.2	41.2	23.5
NR	0.0	0.0	100.0	66.7	0.0	33.3
Total	28.4	11.5	55.4	80.7	16.2	7.8
Wave: Chi-square (p-value)					70.4773 (0.000*)	
**Recording of administered medicines in wards (n; wave one=73, wave two=244)**						
Level of Care	Irregular		No		Yes, regular (annually)	
	Wave one	Wave two	Wave one	Wave two	Wave one	Wave two
HC4	38.5	4.1	46.2	89.6	15.4	6.4
GH	32.4	14.6	46.0	70.8	21.6	14.6
RRH	47.1	29.4	11.8	41.2	41.2	29.4
NR	50.0	0.0	50.0	50.0	0.0	50.0
Total	38.4	7.8	38.4	81.6	23.3	10.7

Wave: Chi-square (p-value)					63.2766 (0.000*)	
Overstock and under stock of certain items (n; wave one=74, wave two=244)						
Level of Care	Irregular		No		Yes, regular (annually)	
	Wave one	Wave two	Wave one	Wave two	Wave one	Wave two
HC4	14.3	11.0	50.0	80.9	35.7	8.1
GH	21.6	18.8	32.4	58.3	46.0	22.9
RRH	29.4	29.4	23.5	29.4	47.1	41.2
NR	16.7	0.0	0.0	50.0	83.3	50.0
Total	21.6	13.5	31.1	72.1	47.3	14.3
Wave: Chi-square (p-value)					63.7041 (0.000*)	

Source: Primary data; *Significant at 5%

3.5.2. Reporting of ADR/AEFI reports to NDA

In a drive to combat medicines use problems, the NDA) recommends HF through clinicians and care givers to report any adverse drug reactions (ADR) and adverse events following immunisation (AEFI) to the NDA, a body mandated to regulate medicines quality in Uganda. Figure 8 below shows that the percentage of HF that are not sending ADR/AEFI reports to the NDA increased from 28.4% in wave one to nearly 60% in wave two. Additionally, the largest percentage of HFs (59.8%) do not report medicines quality issues to the NDA and where reporting is done it’s again the pharmacist (12.3%) who sends the reports. At HC4 level, nearly ten in a hundred dispensers report medicine quality issues to NDA.

Figure 8: Reporting of ADR/AEFI Reports and Medicines Quality Issues to NDA

3.5.3. MTC Interventions through CMEs

MTCs are pivotal in promoting appropriate medicines use in HF. One of the channels through which this can be done is by carrying out interventions and conducting continuous medical education (CMEs). Table 15 below shows a declining trend in the percentage of HF MTCs conducting these interventions. At the HF level, NRs (wave one=33.3, wave two=83.3%), RRHs (wave one=70.6, wave two=82.4%), and GHs (wave one=44.4%, wave two=70.8%), had the highest percentage of MTCs advising health workforce and administration on appropriate medicines use. The percentage of MTCs at HC4 level doing this has reduced from 42.9% in wave one to only 21.6% in wave two.

Across all the interventions studied, RRHs, NRs, and GHs recorded significant improvement between the two waves unlike their counterparts at HC4 level. The poor performance at HC4 skewed the overall scores for all the interventions downwards.

Table 15: MTC Interventions through CMEs

MTC Interventions	HC4 (%)		GH (%)		RRH (%)		NR (%)		Total (%)	
	Wave one	Wave two	Wave one	Wave two	Wave one	Wave two	Wave one	Wave two	Wave one	Wave two
Advise medical, pharmacy and administrative staff on appropriate medicine use	42.9	21.6	44.4	70.8	70.6	82.4	33.3	83.3	49.3	37.2
Restrictions (withdrawal of injectables from OPD)	28.6	12.3	30.6	50.0	47.1	76.5	50.0	33.3	35.6	24.8
Conduct pharmacovigilance activities	21.4	7.6	38.9	60.4	70.6	82.4	50.0	66.7	43.8	24.8
Monitor the use of standard treatment guidelines	35.7	12.9	36.1	45.8	52.9	76.5	0.0	33.3	37.0	24.4
Identify medicines use problems	35.7	18.1	36.1	56.3	52.9	82.4	66.7	66.7	42.5	31.4
Design and implement antimicrobial stewardship activities	0.0	4.1	16.7	27.1	41.2	76.5	33.3	50.0	20.6	14.9
Conduct appropriate research on medicine use	0.0	0.6	5.6	8.3	23.5	58.8	0.0	33.3	8.2	7.0
None	50.0	69.0	44.4	16.7	5.9	5.9	33.3	33.3	35.6	53.3
Others specify	0.0	5.9	0.0	6.3	0.0	0.0	0.0	0.0	0.0	5.4

Source: Primary data

The study further explored whether the MTCs documented any intervention activities carried out within the HF. Table 17 below shows the percentage of MTCs documenting their interventions reduced from 50.7% (37/73) in wave one to 35.8% (86/240) in wave two. MTCs should be encouraged to document any interventions they conduct. This should provide a basis for measuring their effectiveness and future performance. Documents that provide justification for evidence-based decision making and will from the HF administration.

Table 16: Documentation of MTC interventions conducted through CMEs and mentoring

Category	Wave one		Wave two	
	Freq	%	Freq	%
Yes, in hard copy form	16	21.9	44	18.3
Yes, in soft copy form	11	15.1	19	7.9
Yes, in both hard and soft copy	10	13.7	23	9.6
No	36	49.3	154	64.2
Total	73	100.0	240	100.0

3.5.4. Monitoring of Medicine Use

Table 17 below shows that the percentage of MTCs that always monitor prescriptions at OPD increased across all levels of care. Overall, the percentage of MTCs that monitor prescriptions at OPD increased from 6.8% in wave one to 13.6% in wave two. RRHs (wave one=11.8%, wave two=47.1%) recorded the highest percentage of MTCs that always monitor prescriptions at OPD. The percentage of HF MTCs that always monitor adherence to STG for specific conditions remained the same at around 13.6% in both waves. RRHs (wave one=17.7%, wave two=47.1%) recorded the highest percentage of MTCs that always monitor adherence to STG for specific conditions. Finally, the findings indicated a downward trend in the percentage of MTCs monitoring changes and adherence to prescription practices from 40.9% in wave one to 23.1% in wave two. The study established a significant relationship between the level of care for the HFs and the rate of monitoring medicine use evaluations by the MTCs (p-value=0.00<0.05)

Table 17: Monitoring of Medicine Use

MTC monitor prescriptions at OPD (n; wave one=74, wave two=242)						
Level of care	No		Yes, always		Yes, sometimes	
	Wave one	Wave two	Wave one	Wave two	Wave one	Wave two
HC4	57.1	82.0	0.0	6.4	42.9	11.6
GH	46.0	38.3	8.1	27.7	46.0	34.0
RRH	17.7	23.5	11.8	47.1	70.6	29.4
NR	33.3	50.0	0.0	16.7	66.7	33.3
Total	40.5	68.6	6.8	13.6	52.7	17.8
level of care: Chi-square (p-value)					69.8192 (0.000*)	
MTC monitoring of adherence to STG for specific conditions (n; wave one=74, wave two=242)						
Level of care	No		Yes, always		Yes, sometimes	
	Wave one	Wave two	Wave one	Wave two	Wave one	Wave two
HC4	50.0	80.8	7.1	8.1	42.9	11.1
GH	47.2	47.9	16.7	20.8	36.1	31.3
RRH	11.8	17.7	17.7	47.1	70.6	35.3
NR	33.3	66.7	0.0	16.7	66.7	16.7
Total	38.4	69.6	13.7	13.6	48.0	16.9
Level of care: Chi-square (p-value)					63.4888 (0.000*)	
MTC monitoring of changes and adherence to prescription practices (n; wave one=74, wave two=242)						

	Wave one		Wave two	
Level of care	No	Yes	No	Yes
HC4	71.4	28.6	86.1	14.0
GH	64.7	35.3	58.3	41.7
RRH	47.1	52.9	41.2	58.8
NR	33.3	66.7	66.7	33.3
Total	59.2	40.9	77.0	23.1
Level of care: Chi-square (p-value)		37.0321 (0.000*)		

Source: Primary data; *Significant at 5%

3.6. Factors Influencing the Performance of MTCS in HFs

The study conducted inferential analysis to establish the factors affecting the functionality of MTCs in HFs in Uganda. Functionality status was the outcome variable in the analysis below. The predictor variables included level of care, nature of HF, availability of guidelines, ToRs, functionality of subcommittees, officially appointed members, number of MTC members, members being active, availability of a work plan and budget. An ordered logistic regression model was estimated using panel data techniques, specifically a random effects model utilising subject specific approaches with the aim to estimate subject specific effects. See analytical framework in the methods section for more details. During model estimation, for each categorical variables in the model, one category was dropped to avoid a dummy variable trap, but essentially to cater for multicollinearity. The overall model was significant at the 95% level of confidence (p-value=0.000<0.05). This means that all the independent variables jointly significantly influence the MTC functionality. The random effects model did not demonstrate high variability between waves (p-value=0.3158>0.05) but was still preferred over the ordered logit model due to the need to account for individual level effects.

Table 18 below shows the estimated net effect and the magnitude of the predictor variables on the functionality status of MTCs. Level of care (p-value<0.05), availability of guidelines (p-value<0.05), availability of subcommittees (p-value<0.05), and MTC members being active (p-value<0.05), where identified as the variables significantly affecting MTC performance.

NRs (OR=43.2) GHs (OR=9.6), and RRHs (OR=8.9) had higher odds of having functional MTCs compared to HC4s. Additionally, having an AMS subcommittee in a HF is 11 times more associated with having a functional MTC than with a partially functional MTC and below. HFs with a functional supply chain subcommittee were nearly seven times (OR=6.5) more likely to have a functional MTC than those with a partially functional MTC and below. MTCs with all members active were sixteen times more likely to have their MTCs fully functional than having part partially functional MTC and below compared to those whose members are sometimes active (OR=9.6) and those with no active members. Some variables produced wide confidence intervals (CI) implying less precision in such variables, specifically attributed to higher variability in the data. Usually, very wide confidence intervals are a result of a small sample size; however, in this study, we believe that a sample size of 265 panels was adequate. Estimation methods are usually another common point of encumbrance; however, the nature of our data dictated the estimation method, and we believe that with all other possible methods tested, using ORM and random effects was the best approach.

Table 18: Determinants of MTC functionality in public HFs

Functionality status	Adjusted Odds Ratio (95% CI)	P-value
Level of care		
HC4”	1	
GH	9.6 [2.745, 33.58]	0.000*
RRH	8.9 [1.461, 54.081]	0.018*
NR	43.2 [3.817, 488.288]	0.002*
Nature of HF		
None teaching HF”	1	
Teaching HF	2 [0.618, 6.71]	0.243
Availability of guidelines		
MTC guidelines (Yes)	1 [0.404, 2.282]	0.927
MTC guidelines (No) ”	1	
PV guidelines (Yes)	2 [0.925, 4.513]	0.077**
PV guidelines (No) ”	1	
AMS guidelines (Yes)	3.1 [1.012, 9.5]	0.048*
AMS guidelines (No) ”	1	
National STG (Yes)	0.6 [0.212, 1.842]	0.394

National STG (No) ”	1	
EMHS list (Yes)	1.8 [0.658, 4.712]	0.26
EMHS list (No) ”	1	
ToR (Yes)	0.4 [0.112, 1.167]	*0.089***
ToR (No) ”	1	
Subcommittees		
PV subcommittee (Yes)	2.2 [0.722, 6.841]	0.164
PV subcommittee (No) ”	1	
AMS subcommittee (Yes)	11 [3.137, 38.62]	0.000*
AMS subcommittee (No) ”	1	
Supply chain subcommittee (Yes)	6.5 [2.142, 19.632]	0.001*
Supply chain subcommittee (No) ”	1	
Research subcommittee (Yes)	0.1 [0.015, 0.58]	0.011*
Research subcommittee (No) ”	1	
Number of MTC members	1 [0.935, 1.103]	*0.712*
Whether MTC members are active		
No”	1	
Yes, some members	9.6 [1.637, 55.75]	0.012*
Yes, all members	16.3 [2.531, 105.44]	0.003*
MTC members officially appointed		
No”	1	
Yes, some members	0.7 [0.177, 3.116]	0.683
Yes, all members	2.2 [0.743, 6.714]	0.152
Workplan (Yes)	1.5 [0.756, 3.036]	*0.242*
Workplan (No) ”	1	

* Significant at 5%, ** Significant at 10%; CI=95% Confidence Interval; ” =Base category

20 below presents the predicted chance of having functional MTCs in HFs. The interventions of NMS, MoH and implementing partners increased the probability of at least having an MTC in place from 22% in wave one to 45% in wave two. This is attributed to the significant number of HC4 MTCs that were established but not yet operational. Overall, the probability of having fully functional MTCs is still low at 25%. This is attributed to the poor performance at HC4 level, otherwise, NRs (73%), RRHs (66%) and GHs (46%) have high chances of having fully functional MTCs. Drastic and focused activities in terms of capacity building and benchmarking should be accelerated at HC4 level. The goal is to use MTCs to combat irrational medicines use in the health care service delivery system. All service delivery points should move at the same pace where possible, by ensuring that the efforts to provide relevant

guidelines, activation of MTC subcommittees, and encouraging full participation of members are accelerated.

Table 19: Predicted probabilities of MTC functionality

Probability of MTC functionality by Wave					
		Wave Number			
	Wave One	Wave Two	Total		
Pr (MTC available but not functional)	0.218	0.446	0.399		
Pr (MTC, partially functional)	0.492	0.321	0.356		
Pr (MTC, fully functional)	0.29	0.234	0.245		
Probability of MTC functionality by level of care					
	HC4	GH	RRH	NR	Total
Pr (MTC available but not functional)	0.600	0.115	0.049	0.009	0.399
Pr (MTC, partially functional)	0.344	0.422	0.296	0.259	0.356
Pr (MTC, fully functional)	0.057	0.462	0.655	0.732	0.245

4.0. Discussion

This study provides the current state of the functionality of MTCs in HFs in Uganda with comprehensive insights into the factors influencing the functionality of MTCs in addition to evaluating the MTC structure, their operations and the MTC interventions. The study found a significant improvement in the prevalence and functionality of MTCs between the two waves under review, moving from 8.3% at the inception of the revitalisation program in 2023 to 20.8% after one year of intervention in 2024. WHO recognizes the establishment of MTCs as one of the strategies to promoting rational medicines use in the health facilities. Medicines use problems are a global challenge that cause ineffective treatment, adverse drug events as well as waste of resources. MTCs are charged with the responsibility of combating prescribing not in accordance with standard treatment protocols, promote good dispensing practices to avoid medication errors, minimise patients' lack of knowledge about dosing schedules, and provide guidance to patients not adhering to dosing schedules and treatment [4]. The outcome of this is to ensure that the principles of drug acquisition, management and use are achieved.

The MTC concept was introduced into the Uganda's public healthcare system in the early 2000s, but their functionality has largely been microscopic [1]. In 2023, National Medical Stores (NMS), MOH and other implementing partners embarked on a process to revitalize the performance of MTCs in HFs. The focus was to have the MTC structures in place, rejuvenate MTC operations, and have targeted interventions. A baseline assessment study was conducted to establish the functionality of these committees, and this indicated suboptimal functionality characterised by lack of commitment from MTC members where they existed in addition to minimal support from the HF administration as well as knowledge gaps in MTC operations. Similarly, Kimbowa etl., (2023) conducted a study on the "role of medicines and therapeutics committee's structure in supporting optimal antibacterial use in hospitals in Uganda", in which they found 31.3% (21/67) presence of MTCs in the hospitals (RRH and GH) in Uganda [34]. Their study did not explore the opportunity to investigate the level of MTC functionality as it is understood that in many instances, since the MoH embarked on the process to establish MTCs in HFs, the majority remained on paper or sub optimally functioned, a problem that was not just alien only to Uganda. Rwanda, Ethiopia and many other LMICs face a similar challenge [16,34,35]. After one year of intervention, this study used panel data strategies to provide up-to-date information regarding the functionality of MTCs in HFs in Uganda, evaluating the MTC structure, its operations and impact. The findings provide a significant improvement since the beginning of the intervention, an indication of hope if sustainability mechanisms are put in place.

The WHO recommends that an MTC should be diverse in nature, comprising of professionals from all disciplines. This offers the necessary technical competencies in discussing drug use issues and offers an opportunity to educate members in areas that are not in line with their expertise[4]. Additionally, the MoH MTC manual recommends that an MTC should have between 12 to 15 MTC members[31]. The median number of MTC members in wave one and

wave two was 13 and 15 respectively, demonstrating strong adherence to the recommended number in the MoH MTC manual. The median number in the study findings differed considerably from what Kimbowa *et al.* (2024), found in their study. This indicated a 10 MTC membership. Kiruyi *et al.* (2023) in a systematic review of the literature on the MTC structure and roles in LMIC found a median number ranging between 8 to 25, although there is a lack of an internationally recommended number of MTC members other than encouraging such committees to be multidisciplinary [8], even in developed economies the median number of MTC members range from as low as 2 members whereas others boast up-to 40 members [10]. It's worth noting that the findings from this study are more generalizable since it covered a full sample of all public high-level HFs. This is an indication that while establishing the MTC structures during reactivation phase, the level of participation from the health workforce in the HFs has improved. This was also evident in the level of participation in MTC activities which improved from 25.7% in wave one to 51.2% in wave two.

The presence of MTC subcommittees is critical if the MTCs are to operate optimally. Subcommittees many a times perform the cardinal role of the MTC. This gives the MTC a leverage to perform its oversight role. The subcommittees are expected to hold more frequent meetings than the main MTC. In this study, the availability of subcommittees significantly improved from 64.5% in wave one to 93% in wave two. These were at varying levels of functionality, with NRs and RRHs more likely to have functional subcommittees compared GHs and HC4s. The most prevalent and functional subcommittees were the supply chain subcommittee and the AMS subcommittee. The supply chain subcommittee is mandated to spearhead inventory management practices in the HF while the AMS subcommittee is charged with the responsibility of assisting the main MTC in the management of antimicrobials [31]. In many countries, the AMS and the supply chain subcommittee are the most prevalent, although the infection prevention and control (IPC) is also more common [8,36]. For many

years, the most vibrant subcommittee has been the supply chain subcommittee. This is due to the need to ensure appropriate medicines selection, quantification and management as aligned in the national medicines policy 2015 [34]. In recent times, the AMS subcommittee has gained more attention in Uganda due to the interventions from partners like the MSH program under MTaPS whose primary goal was to control antimicrobial resistance [1]. The current intervention by the MoH, NMS and other partners, establishment and functionalization of the AMS subcommittee is at the forefront. The study found a statistically strong linkage between the AMC and the supply chain subcommittee and the functionality of MTCs. The research subcommittee was also significant, but not as much compared to the other two subcommittees. The findings in this study are consistent with Kimbowa *etl.,* (2024), who found high presence of AMS and supply chain subcommittees and a moderate presence of the PV and other subcommittees in hospital MTCs in Uganda [34]. In relations to other countries, the presence of the AMS subcommittee was however lacking in many Nigerian hospitals, where, twenty five percent of hospitals had this committee and the most prevalent subcommittee was instead the IPC subcommittee, found in seventy five percent of the hospitals [16].

The MTC, through their subcommittees are tasked with the responsibility of developing policies and guidelines to guide medicines management and use. For example, developing the policy by the AMS subcommittee to steward the use of antimicrobials in the HFs or development of an antibiogram. Worryingly, the development of policies and guidelines as outlined in the MTC manual 2018 dropped sharply from what was observed in wave one. Most MTCs have not developed any policies to curb the growing concern of drug promoters in HFs. Whereas it's important for the prescribers to learn about any new pharmaceuticals/molecules on the market, the introduction of such medicines should be through a regulated framework, most importantly guided by the institution medicines list commonly known as the hospital drug formulary [31]. The MTCs where they exist should oversee the selection of EMHS necessary

for use in their setting by identifying and profiling medicines pertaining to the healthcare needs of their catchment population and ensuring their rational use within the HF and the community [6]. The study found a significant decrease in the percentage of HF MTCs that had developed an institutional EMHS list by around ten percentage points from 22.2% in wave one to only 9.9% in wave two. This shouldn't be the case especially given the fact that one of the strategic directions of the healthcare system in Uganda is to fight against misuse of medicines. This is contrary to findings from many studies on MTCs, where one of the topmost prioritized policies is the hospital drug formulary in both developed and developing economies [16,23,36].

MTCs are charged with the responsibility of spearheading the promotion of rational medicine use in HFs. This should be through ensuring proper diagnosis, prescribing safe medication, selecting appropriate medicines in their correct dosage and duration, and providing patients with correct information as well as evaluating treatment responses. MTCs may therefore be understood as a platform used by the multidisciplinary professionals in a HF to discuss drug use and drug use problems [37]. Medicines use audits, surveys, and interventions through CMEs are among the activities MTCs should prioritize for implementation. The study found significant progress in RRHs, NRs, and GHs, however, minimal progress in ensuring that these key activities are conducted was observed in HC4s. Antimicrobial Resistance (AMR) remains a global challenge, posing threats to human health and existence [38]. In Uganda, this problem is accelerated by lack of knowledge, overuse, misuse, and abuse of antimicrobials such as non-adherence to dosage, self-medication, and purchase of drugs without prescription [39]. Therefore, the need to design and implement interventions that provide stewardship towards regulating and controlling the use of antimicrobials to curb AMR is one of the drivers for advancing health knowledge to protect the current and future generations [38].

The national standard treatment guidelines provide a framework for ensuring adherence to treatment standards in Uganda's healthcare service delivery system by providing and

promoting the required information for diagnosis and management of common conditions. In the HFs, MTCs are pivotal in promoting these standards. The MoH MTC manual 2018 encourages MTCs to monitor prescriptions at OPD, monitor adherence to STG for specific conditions, and monitoring of changes and adherence to prescription practices, where the goal is to curb overuse of antibiotics, indiscriminate use of injectables, polypharmacy among others. Findings in our study indicate that this is still far-fetched, especially in levels of care that offer less specialized services and whose capacity to manage these activities is vacillated.

The study was comprehensive in nature, covering all public higher level health facilities in the country. Therefore, unlike other studies that have been conducted in the same area in Uganda whose scope has been limited both in population and in content, our study presents more generalizable findings appeasing the literature space for knowledge extraction. Additionally, while our study established significant improvements in the presence of MTCs and availability of structures, drastic and focused activities in terms of capacity building and benchmarking should be accelerated in conducting such activities such as carrying out prescription audits, medicines use evaluations and point prevalence surveys with a focus on combating medicines use problems and poor prescribing patterns, thereby combating irrational medicines use in the health care service delivery system. All HFs should move at the same pace where possible, by ensuring that the efforts to provide relevant guidelines, and encouraging full participation of members are promoted. Interventions should focus on strengthening the MTC subcommittees by revitalising them and establishing them where they are lacking.

5.0. Conclusions

The study established a significant improvement in the functionality of MTCs in public HFs. This is attributed to the interventions by the MOH, NMS, implementation partners and the commitment from the HF staff and management. Whereas a limited number of MTCs are fully functional, the status has drastically changed with almost no HF without an MTC regardless of

the functionality status. The operating strategies usually start with setting up the MTC structure. This has been greatly achieved across all levels of care, including the HC4s that were brought on board in the space of one year. There was less improvement in the MTC operations, especially carrying out activities that drive the fight against inappropriate medicine use in HFs. Interventions in medication use stewardship should be strengthened at all levels of care. There is demonstrated hope for sustainability. The results indicated significant improvement in the funding of MTC activities by the HFs, especially high-level HFs.

6.0. Acknowledgement

NMS would like to express sincere thanks to all who participated in the data collection exercise. Special thanks go to the Ministry of Health (MoH) and the Department of Pharmaceuticals and Natural Medicines (DPNM) for providing guidance. Hospital directors, medical superintendents, pharmacists, HC4 In-charges, MTC chairpersons, MTC secretaries, and MTC members in public hospitals who provided valuable information during the interviews.

We also wish to extend our sincere gratitude to NMS pharmacists in the regions who provided guidance to all stakeholders that were interviewed.

7.0. References

[1] MSH. Improving appropriate medicine use through hospital medicines and therapeutics committees. 2020.

[2] Monégat M, Sermet In collaboration with Marc Perronnin C, Rococo E. Polypharmacy: definitions, measurement and stakes involved. Review of the literature and measurement tests. 2014.

[3] WHO. WHO Policy Perspectives on Medicines-Promoting rational use of medicines: core components WHO Policy Perspectives on Medicines. Geneva: 2002.

[4] Organization WH, Holloway K, Green T, Carandang E, Hogerzeil H, Laing R, et al. Drug and therapeutics committees A practical guide with contributions from. 2003.

[5] Matlala M, Gous AGS, Meyer JC, Godman B, Rosenkranz B, Lofts A, et al. Formulary Management Activities and Practice Implications Among Public Sector Hospital Pharmaceutical and Therapeutics Committees in a South African Province 2020;11:1. https://doi.org/10.3389/fphar.2020.01267.

[6] Mordujovich-Buschiazzo P, Cañás M. Manual of Procedures for the Pharmacotherapy-Medicines Selection Committees. n.d.

[7] Durán-García E, Santos-Ramos B, Puigventos-Latorre F, Ortega A. Literature review on the structure and operation of Pharmacy and Therapeutics Committees. Int J Clin Pharm 2011;33:475–83. https://doi.org/10.1007/s11096-011-9501-6.

[8] Kiruyi S, Nagadya C, Walusimbi D, Mukooza EK, Higenyi E, Rajab K. Drug and Therapeutics Committee Structure and Roles in Hospitals: A Scoping Review. Texila International Journal of Public Health 2023;11. https://doi.org/10.21522/TIJPH.2013.11.04.Art006.

[9] Thuè PA, Harder S, Steio€ A. Pharmacoepidemiology and Prescription: Structure and activities of hospital drug committees in Germany. n.d.

[10] Fijn R, Brouwers JRBJ, Knaap RJ, De Jong-Van den Berg LTW. Drug and Therapeutics (D and T) committees in Dutch hospitals: A nation-wide survey of structure, activities, and drug selection procedures. Br J Clin Pharmacol 1999;48:239–46. https://doi.org/10.1046/j.1365-2125.1999.00001.x.

[11] ANDRE WW. BROWN CWBH. Hospital Pharmacy Committees in England Their Structure, Function, and Development. BritishMedical Journal 1975;1:323–6. https://doi.org/10.1136/bmj.1.5953.323.

[12] Meena DK, Jayanthi M, Ramasamy K. Availability of Key Essential Medicines in Public Health Facilities of South Indian Union Territory: One of the Crucial Components of Universal Health Coverage 2021. https://doi.org/10.7759/cureus.19419.

[13] Bigdeli M, Peters DH, Wagner AK. Medicines in Health Systems: Advancing access, affordability and appropriate use. 2014.

[14] USAID. SIAPS Technical Brief 2 Graphics adapted from Drug and Therapeutics Committees: A Practical Guide. 2017.

[15] Magulu I, Id K, Ocan M, Mukonzo J, Nakafeero M, Id JE, et al. The role of medicines and therapeutics committees structure in supporting optimal antibacterial use in hospitals in Uganda: A mixed method study 2024. https://doi.org/10.1371/journal.pone.0289851.

[16] Fadare JO, Ogunleye O, Obiako R, Orubu S, Enwere O, Ajemigbitse AA, et al. Drug and therapeutics committees in Nigeria: evaluation of scope and functionality. Expert Rev Clin Pharmacol 2018;11:1255–62. https://doi.org/10.1080/17512433.2018.1549488.

[17] Kamba PF, Nambatya W, Aguma HB, Charani | Esmita, Rajab K. Gaps and opportunities in sustainable medicines use in resource limited settings: A situational analysis of Uganda. Br J Clin Pharmacol 2022;88. https://doi.org/10.1111/bcp.15324.

[18] Goruntla N, Ssesanga J, Reddy Bommireddy B, Thammisetty DP, Vishwanathasetty VK, Obiezu J, et al. Evaluation of Rational Drug Use Based on WHO/ INRUD Core Drug Use Indicators in a Secondary Care Hospital: A Cross-Sectional Study in Western Uganda 2023. https://doi.org/10.2147/DHPS.S424050.

[19] Akunne AA, Lam WI, Ezeonwumelu JOC, Ebosie JC, Udechukwu BE. Assessment of Rational Prescribing in General Outpatient Department of Kampala International University Teaching Hospital, Western Uganda. Pharmacology & Pharmacy 2019;10:48–60. https://doi.org/10.4236/pp.2019.101005.

[20] Kanyike AM, Olum R, Kajjimu J, Owembabazi S, Ojilong D, Nassozi DR, et al. Antimicrobial resistance and rational use of medicine: knowledge, perceptions, and training of clinical health professions students in Uganda. Antimicrob Resist Infect Control 2022;11. https://doi.org/10.1186/s13756-022-01186-9.

[21] MoH-Uganda. Uganda National Medicines Policy 2015. 2015.

[22] Stichele RV. Editorial The 'Wise List'-A Comprehensive Model for Drug and Therapeutics Committees to Achieve Adherence to Recommendations for Essential Drugs Among Prescribers? Basic Clin Pharmacol Toxicol 2011;108:221–3. https://doi.org/10.1111/j.1742-7843.2011.00684.x.

[23] Plet HT, Hallas J, Nielsen GS, Kjeldsen LJ. Drug and Therapeutics Committees in Danish Hospitals: A Survey of Organization, Activities and Drug Selection Procedures 2012. https://doi.org/10.1111/bcpt.12028.

[24] Long JScott, Freese Jeremy. Regression models for categorical dependent variables using Stata. Stata Press; 2001.

[25] Greene WH, Hensher DA. Modeling Ordered Choices: A Primer and Recent Developments. 2008.

[26] Rodríguez G. Models for Longitudinal and Clustered Data. 2008.

[27] Hu FB, Goldberg J, Hedeker D, Ft Flay B, Ann Pentz M. Comparison of Population-Averaged and Subject-Specific Approaches for Analyzing Repeated Binary Outcomes. vol. 147. 1998.

[28] Leung DHY, Wang Y-G, Zhu M. Efficient parameter estimation in longitudinal data analysis using a hybrid GEE method. Biostatistics 2009;10:436–45. https://doi.org/10.1093/biostatistics/kxp002.

[29] Agresti A. An Introduction to Categorical Data Analysis Second Edition. n.d.

[30] STATA LONGITUDINAL-DATA/PANEL-DATA REFERENCE MANUAL RELEASE 18. n.d.

[31] Mwebaze, Sheila. Medicine and Therapeutics Committees Manual 2018. 2018.

[32] Truong WR, Hidayat L, Bolaris MA, Nguyen L, Yamaki J. The antibiogram: key considerations for its development and utilization JAC-Antimicrobial Resistance. JAC Antimicrob Resist n.d. https://doi.org/10.1093/jacamr/dlab060.

[33] Su E, Liew DFL, Donnelly J, Elliott RA. Medicines stewardship. Aust Prescr 2023;46:24–8. https://doi.org/10.18773/austprescr.2023.010.

[34] Kimbowa IM, Ocan M, Mukonzo J, Nakafeero M, Eriksen J, Lundborg CS, et al. The role of medicines and therapeutics committees structure in supporting optimal antibacterial use in hospitals in Uganda: A mixed method study. PLoS One 2024;19. https://doi.org/10.1371/journal.pone.0289851.

[35] MoH-Rwanda. Republic of Rwanda Ministry of Health Medicine and Therapeutics Committee Manual (MTC Manual). Kigali: 2022.

[36] Mittmann N, Knowles S. A survey of pharmacy and therapeutic committees across Canada: scope and responsibilities. vol. 16. 2009.

[37] David Shulkin M. Reinventing the Pharmacy and Therapeutics Committee 2012;37.

[38] Abimbola S, Otieno M, Cole J. Reducing the Use of Antimicrobials as a Solution to the Challenge of Antimicrobial Resistance (AMR): Approaching an Ethical Dilemma through the Lens of Planetary Health. Challenges 2021;12:23. https://doi.org/10.3390/challe12020023.

[39] Musoke D, Lubega GB, Gbadesire MS, Boateng S, Twesigye B, Gheer J, et al. Antimicrobial stewardship in private pharmacies in Wakiso district, Uganda: a qualitative study. J Pharm Policy Pract 2023;16. https://doi.org/10.1186/s40545-023-00659-5.

Figure

Click here to access/download;Figure;Figure 1.png

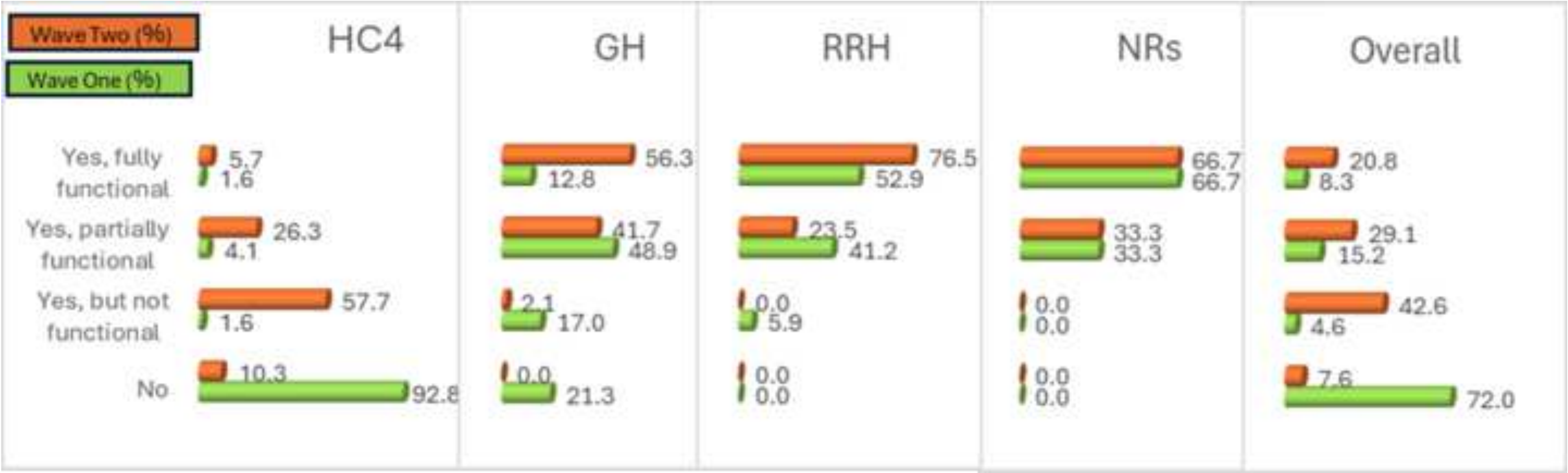

 Click here to access/download;Figure;Figure 2.png

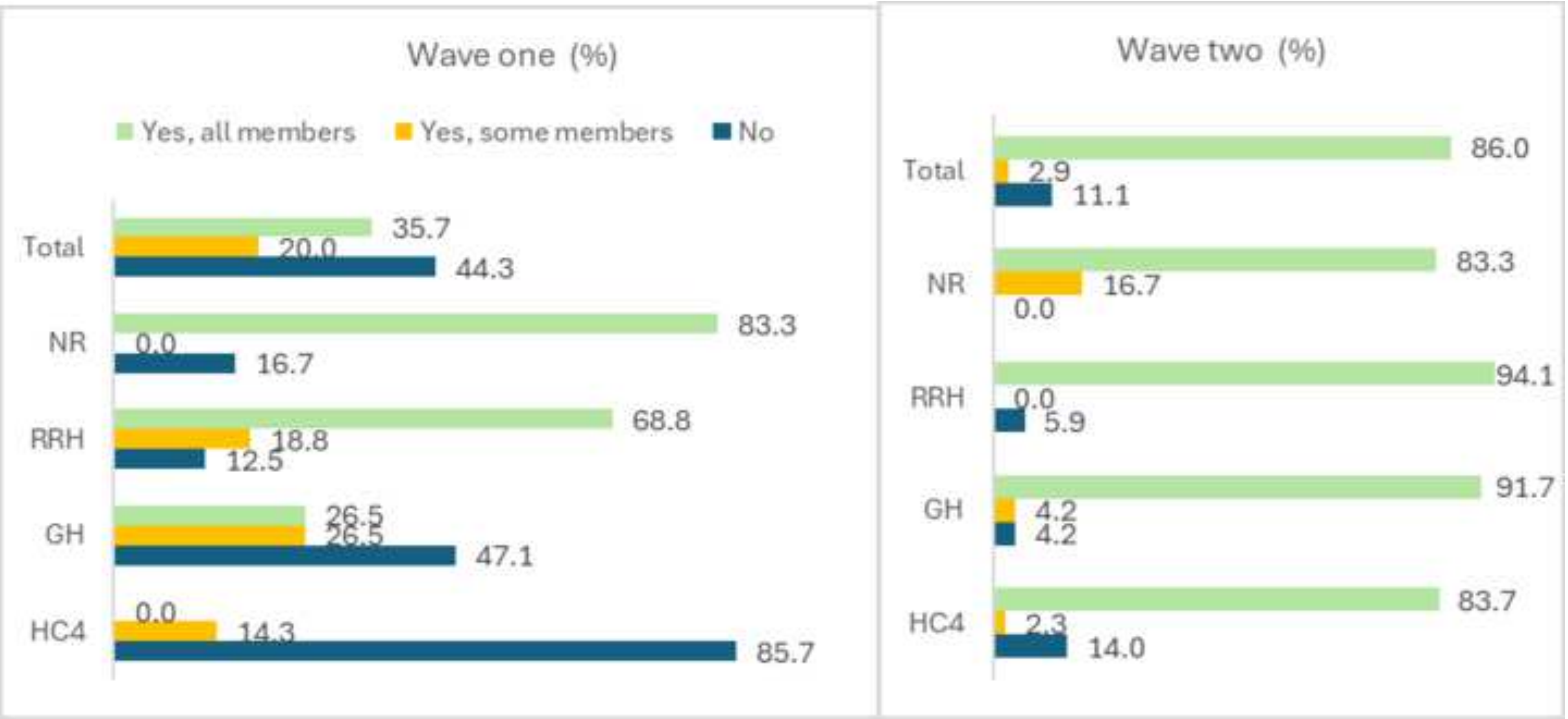

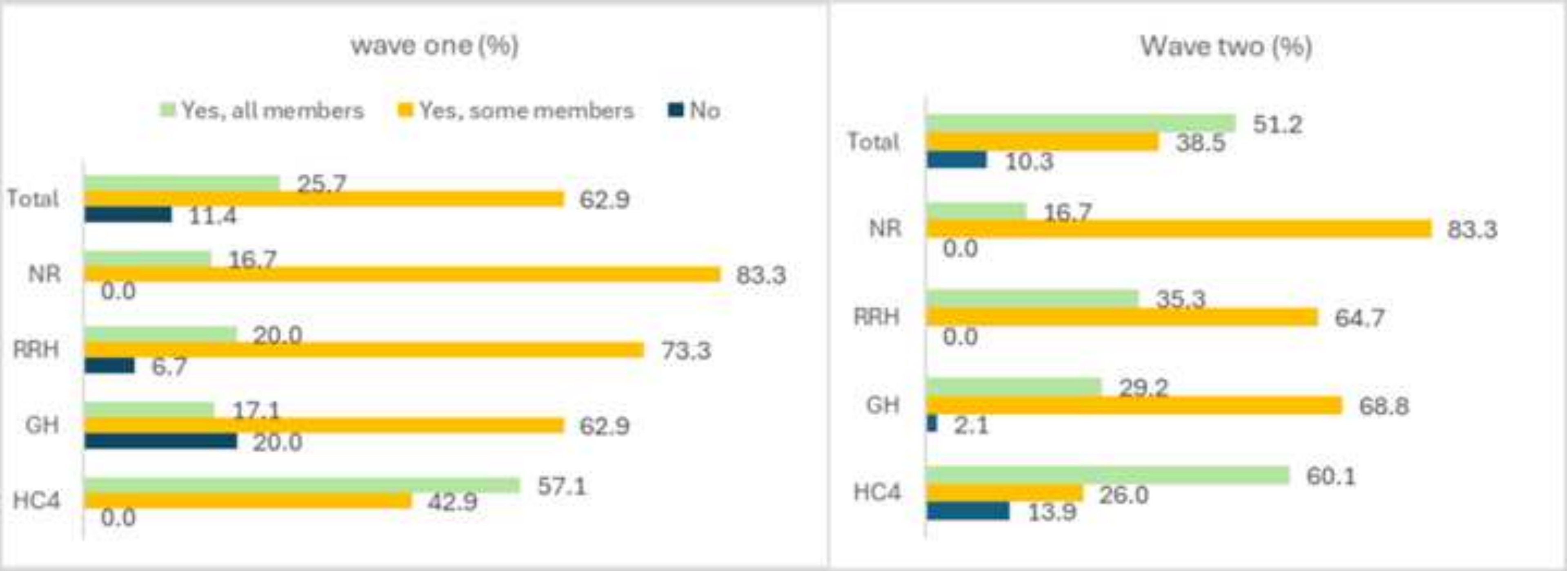
wave one (%)
Yes, all members
Yes, some members
No
Total
25.7
62.9
11.4
NR
16.7
83.3
0.0
RRH
20.0
73.3
6.7
GH
17.1
62.9
20.0
HC4
57.1
42.9
0.0
Wave two (%)
Total
51.2
38.5
10.3
NR
16.7
83.3
0.0
RRH
35.3
64.7
0.0
GH
29.2
68.8
2.1
HC4
60.1
26.0
13.9

Figure

Click here to access/download;Figure;Figure 4.png

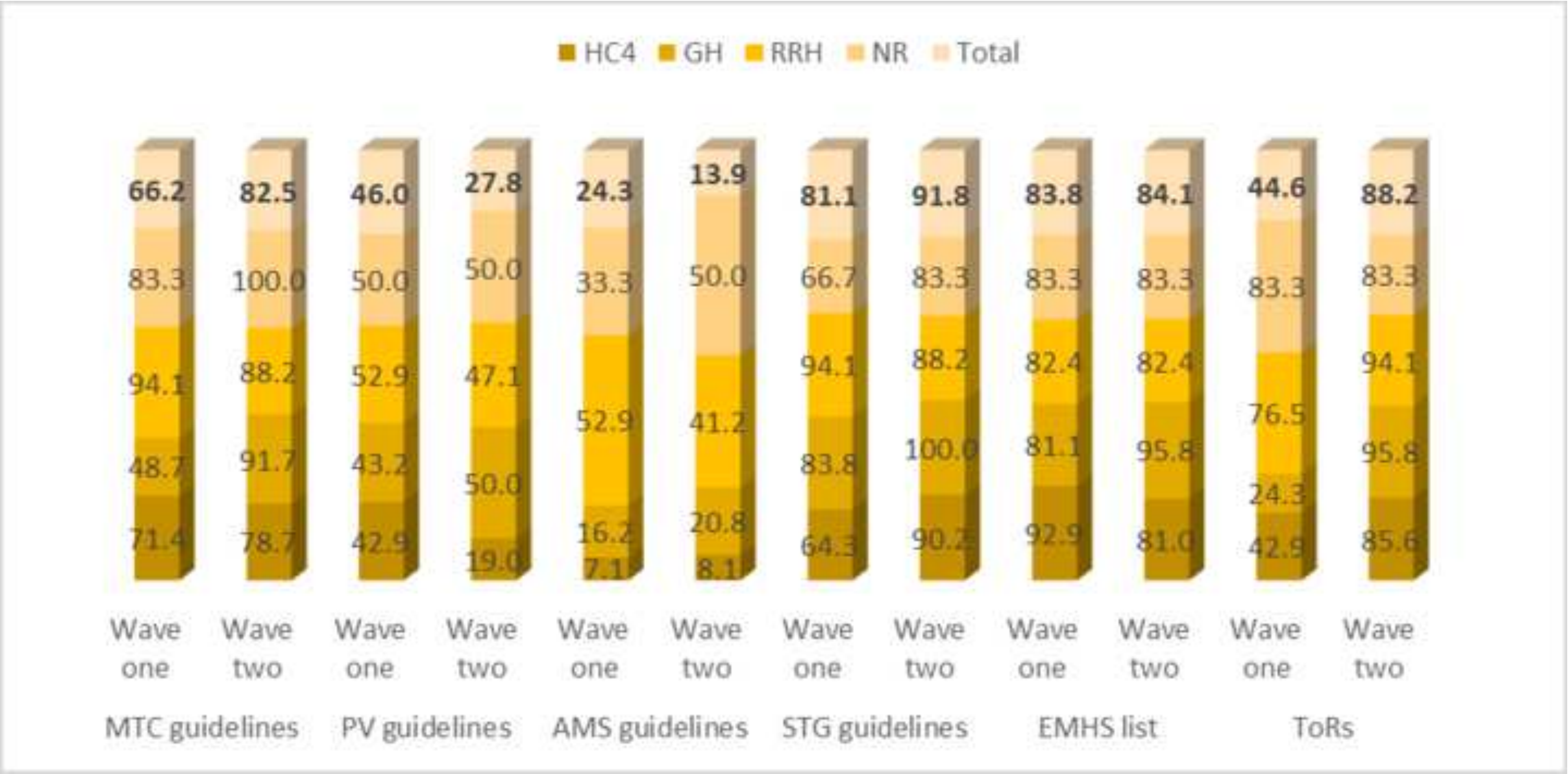

Figure
Click here to access/download;Figure;Figure 5.png

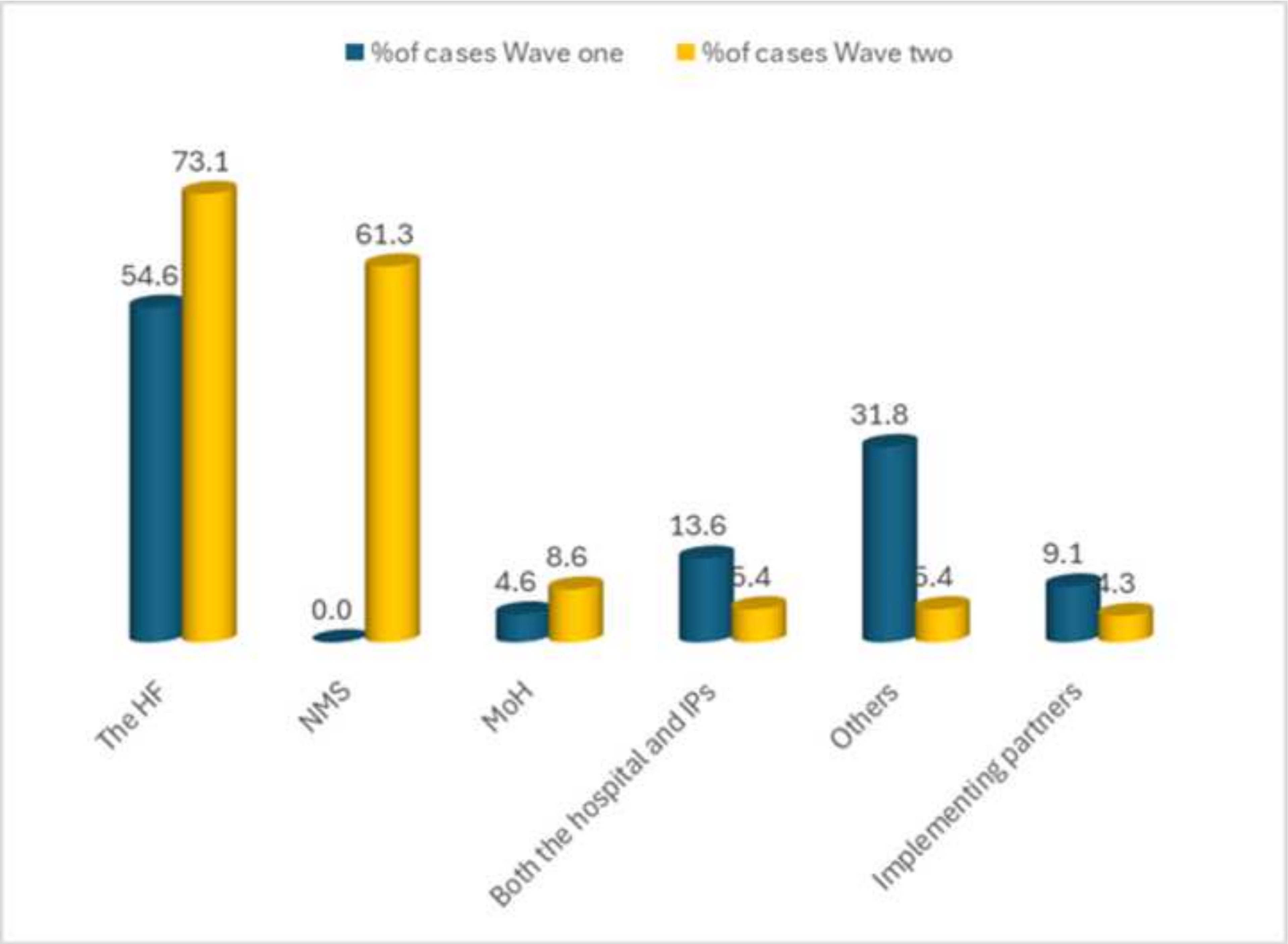

Figure

Click here to access/download;Figure;Figure 6.png

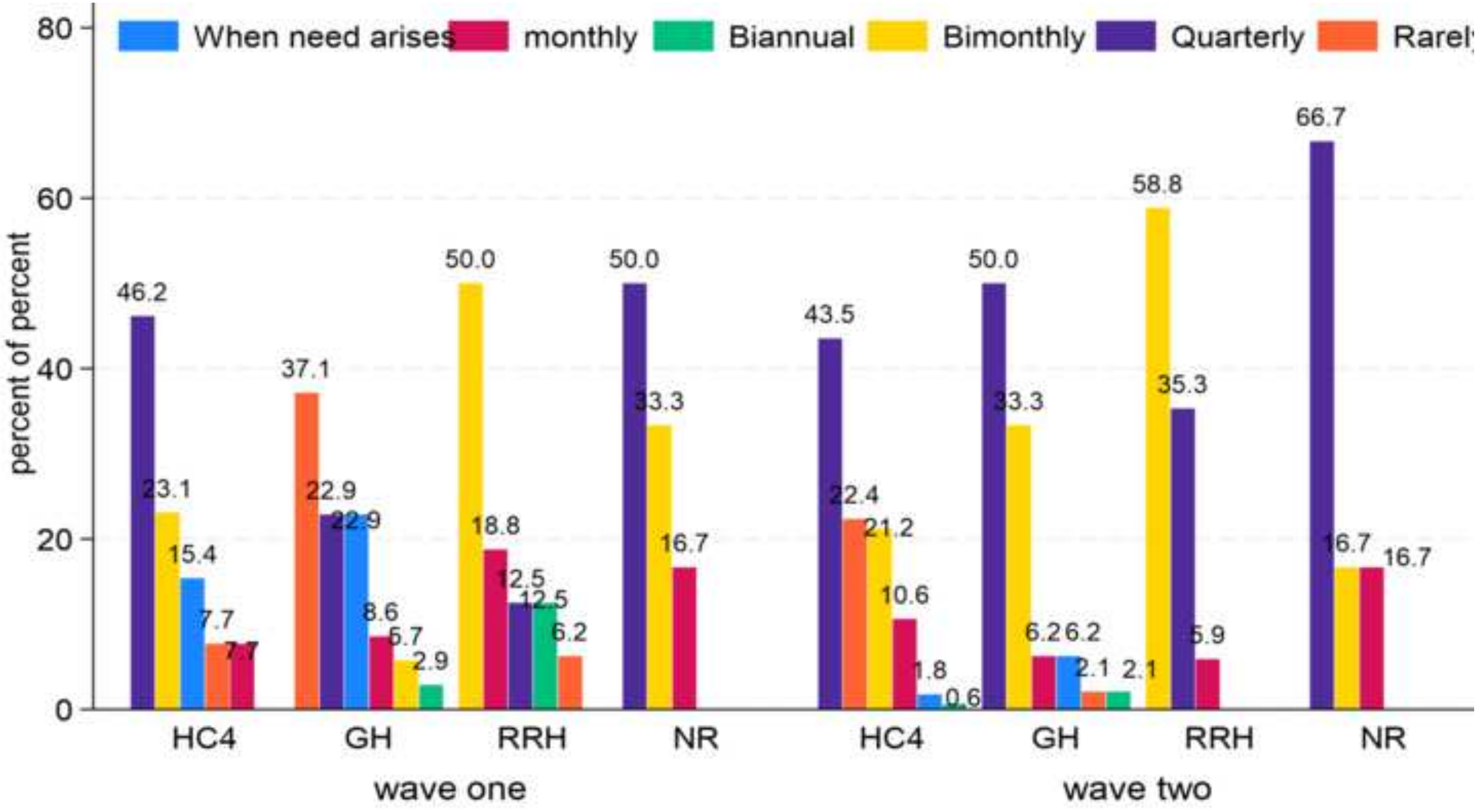

Figure

Click here to access/download;Figure;Figure 7.png

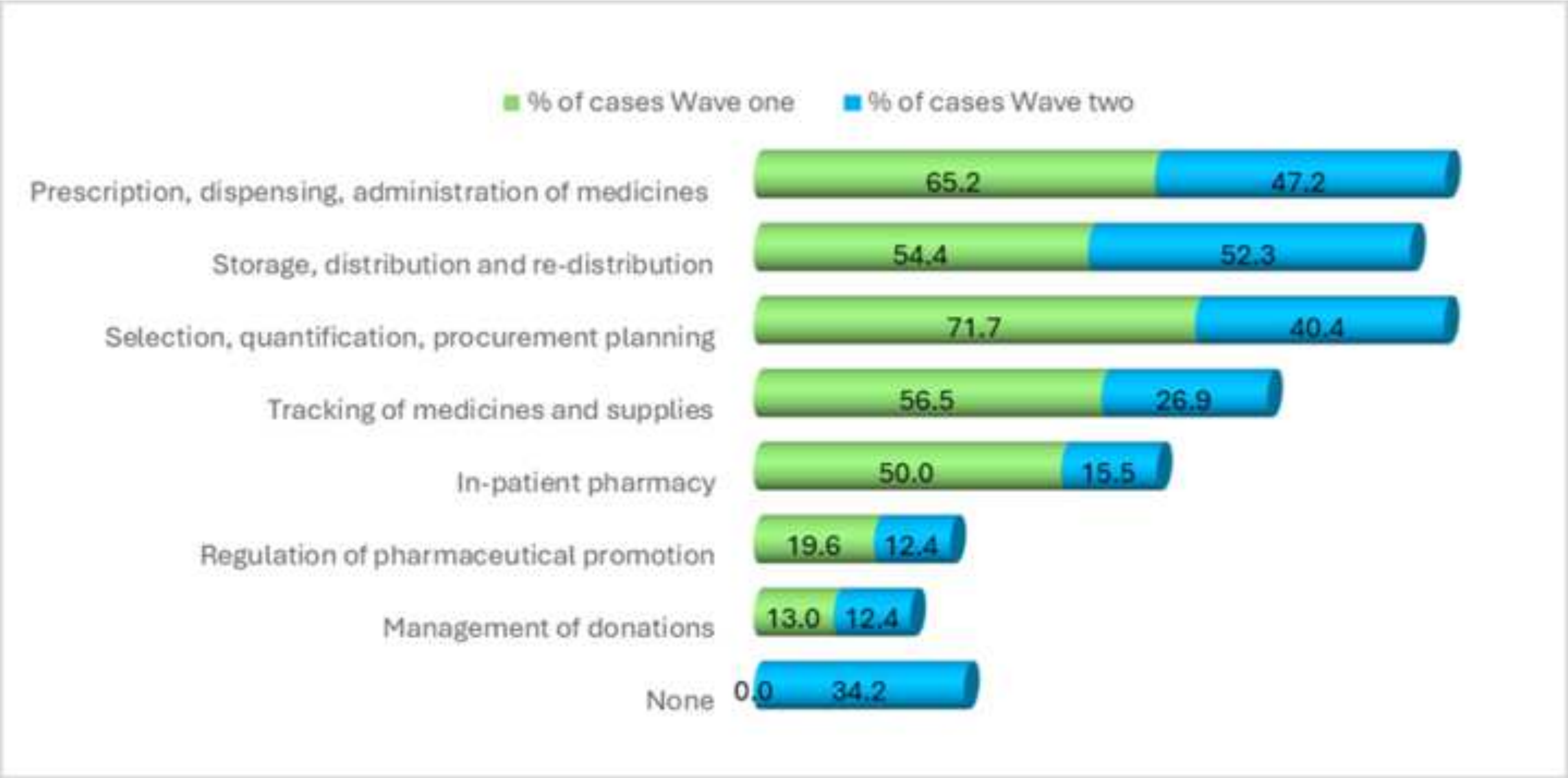

Figure

Click here to access/download;Figure;Figure 8.png

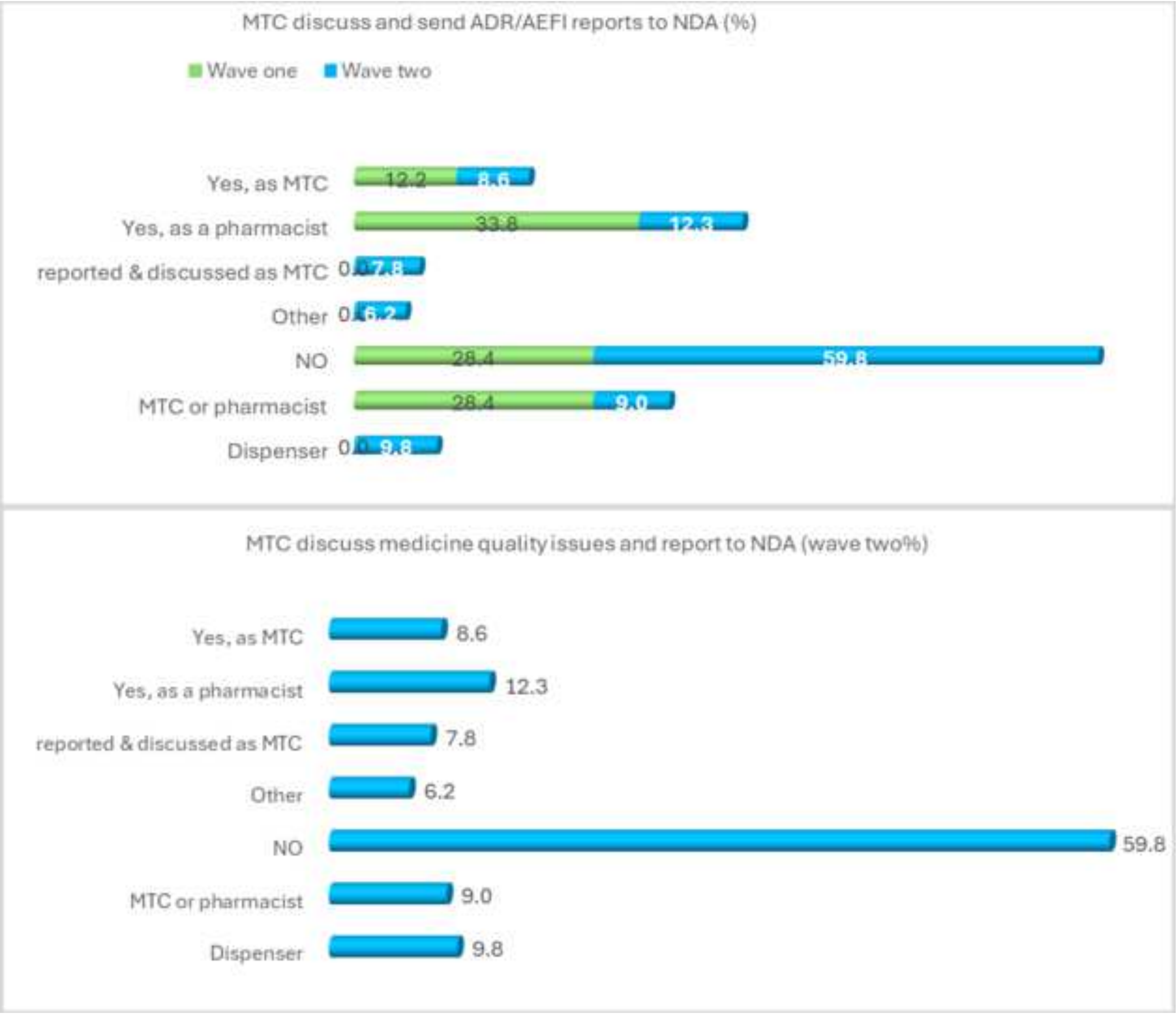

Click here to access/download
Supporting Information
Data Collection Tool.pdf

Click here to access/download
Supporting Information
Model Estimation.log

Click here to access/download
Supporting Information
Authors contribution and other information.docx
