# Supplementary material for: Factors influencing the functionality of medicines and therapeutic committees in public health facilities in Uganda: A longitudinal assessment
Source: PLoS One. 2026 Feb 11;21(2):e0342487. doi: 10.1371/journal.pone.0342487 (PMC12893557; doi:10.1371/journal.pone.0342487)
Supplement: Attachment — Submitted filename: Response to Reviewers.docx [file pone.0342487.s005.docx]

**Factors Influencing the Functionality of Medicines and Therapeutic Committees in Public Health Facilities in Uganda: A Longitudinal Assessment**

Benard Nsubuga

National Medical Stores

Plot 261, Kiwamirembe Road, Kajjansi.

**18^th^ September 2025**

**The Editor**

**PLOS One**

**RE: POINT BY POINT RESPONSE FOR THE RESUBMITTED MANUSCRIPT**

Following reviewing of the above manuscript we hereby submit a revised version after addressing the comments from the editor and the two reviewers. The table below indicates the editor and reviewers’ comments and the corresponding adjustments that were made in the revised version of the manuscript. The entire manuscript has been significantly reduced from 49 pages to 27 pages including references.

**Editor**

| **Comment** | **Page number** | **Action** |
| --- | --- | --- |
| Please ensure that your manuscript meets PLOS ONE's style requirements, including those for file naming | Cover Page | Author names have been rewritten according to the provided guidance by PLOS One. |
| We note that your Data Availability Statement is currently as follows: All relevant data are within the manuscript and in Supporting Information files. Please confirm at this time whether or not your submission contains all raw data required to replicate the results of your study. Authors must share the “minimal data set” for their submission. PLOS defines the minimal data set to consist of the data required to replicate all study findings reported in the article, as well as related metadata and methods | Neutral | Raw data has been provided as a supplementary file in excel form. |
| Please amend either the title on the online submission form (via Edit Submission) or the title in the manuscript so that they are identical. | Neutral | Addressed. |
| Please amend either the abstract on the online submission form (via Edit Submission) or the abstract in the manuscript so that they are identical. | Neutral | Addressed. |
| We note you have included a table to which you do not refer in the text of your manuscript. Please ensure that you refer to Table 1,16 and 19 in your text; if accepted, production will need this reference to link the reader to the Table. | Neutral | This has been addressed. |
| Please include captions for your Supporting Information files at the end of your manuscript, and update any in-text citations to match accordingly. Please see our Supporting Information guidelines for more information |  | Addressed. |
| If the reviewer comments include a recommendation to cite specific previously published works, please review and evaluate these publications to determine whether they are relevant and should be cited. There is no requirement to cite these works unless the editor has indicated otherwise. | Neutral | None. |

**Reviewer Comments**

| **Comment** | **Page number** | **Action** |
| --- | --- | --- |
| The main body of text is 45 pages (excluding references, front page and figures). I know there is no limit to texts for PLOS One, but I think this is much too long for the findings in this study. The background could easily by reduced to 1.5-2 pages | Pages 2-4 | The background section has significantly been reduced from 5 pages to 2.5 pages. |
| I think the results are interesting, but they are not very complex and could be presented in a short article, maxiumum 1/3 of the current text. Select the most important results rather than presenting everything you have | Pages 8-21 | This has been addressed. Only key findings have been retained. |
| Abstract: Rephrase first sentence:” …. irrational use of medicines may constitute 50% of xxxxxx….” or”” of all medicine use globally, aroor”” ofis considered irration….” or similar. Now it is unclear what the 50% are of | Page 2 | World health organization estimates that of all medicines used globally, about 50% are used irrationally. This is mostly attributed to overuse of antibiotics and indiscriminate use of injectables, among other things. |
| The” two waves” – unclear what this is. Repeated measurements? | Page 5 | This study was a panel study, one of the types of longitudinal study design. Longitudinal study design has several types including trend, time series, pooled cross-sectional, panel, cohort etc.  It should be noted that under longitudinal studies, panel assessment and cohort assessments are sometimes referred to as repeated measures, although this is not very correct. The team repeated measures is more used/applicable in experimental designs. Our study was an observational study. |
| Background: Rephrase same sentence as in abstract. | Page 2 | World health organization estimates that of all medicines used globally, about 50% are used irrationally. This is mostly attributed to overuse of antibiotics and indiscriminate use of injectables, among other things. |
| Page 3, first para: Sentence starting with” MTCs were identified….” is part of the methodology, not background. The following sentence:” The aim….” should be at the end of the background section. | Neutral | This sentence has been dropped from the background. Aim of the study has been shifted to the end of the background. |
| Background: Length max 2 pages. No need for e.g. all the history in a research article. | Page 2 | The background has been rewritten and reduced to 2.5 pages. |
| Methodology: Study design is not clear. Is this a repeated cross-sectional study with data collection conducted at two time points? | Page 5 | This was a panel study, one of the types of longitudinal study design. Longitudinal study design has several types including trend, time series, pooled cross-sectional, panel and cohort.  This was a panel study design and data were collected separately for each wave/panel round. |
| When was data collection done? | Page 6 | The study was conducted between January and February 2025. This has been included in the methodology section. |
| Who were the research team that collected data? | Page 6 | The data collection team comprised of 20 pharmacists who were carefully recruited due to the diverse knowledge they possess in supply chain and medicines management. A one-day training workshop was held to equip them with the required interview skills as well as understanding the objective of this study and how to administer questions in the survey tool. |
| Statistics – this should be shortened to one paragraph – no need for formulas in a research article. | Neutral | All formulas have been dropped. |
| Length of methodology max 2 pages | Pages 5-7 | The methodology has been significantly reduced from seven to three pages.  Whereas 2 pages were proposed. It was also necessary to clearly articulate the estimation process at multivariate level for readers who would wish to replicate this modelling approach on ordered outcomes in panel assessments. This regrettably added a third page in the methodology. |
| Results: Too long! E.g. the first paragraph is completely unnecessary. In addition to unnecessarily mentioning what sections will follow, you repeat the methodology. Section 3.2.1 has a discussion about what statistical method was chosen – delete! | Pages 8-20 | This has been addressed. The paragraph that was unnecessarily mentioning subsections in the results has been dropped. |
| You mix in discussion in the results, e.g. page 17, first paragraph, sentence starting “this means….”. Discussion should not be put in results. | Pages 8-20 | Discussion of findings in the results section has been dropped |
| I think you need to reduce the results to maximum 1/4 of what you have here. The text is too long and difficult to follow. If you want to publish all these results, I suggest you split them into different articles. As it is now, the number of figures and results make it impossible to understand what is important and the reader is lost. | Pages 8-20 | Only most important findings have been retained.  Unnecessary text has been dropped while presenting the findings. |
| Discussion The discussion should discuss your findings. At it reads now, it is a general overview of the status of MTCs in Uganda and elsewhere, but you have not discussed your findings in relation to this. The discussion should also be shortened but maybe only by about ½ of what you have now. However, the most important issue here is that you need to discuss your results. And the conclusion should be a summary statement of the discussion, not something new. | Pages-20-24 | Most parts of the discussion section have been rewritten focusing on discussing key findings. The discussion section has been reduced to only 3.5 pages. |
| Reviewer #2: The authors have submitted a thesis. This is not written for a journal. The authors need to re-write the manuscript in the form of a journal articles. The introduction, methods, results, and discussion are too long, they appear to be directly lifted from a project thesis. All the sections need to be re-written and shortened. | Pages 1-25 | All the sections in this manuscript have been significantly reduced to make it fit enough for a journal article. The entire manuscript has reduced from 49 pages in the first submission to only 27 pages including references in the revised version. |
