# Supplementary material for: Factors influencing the functionality of medicines and therapeutic committees in public health facilities in Uganda: A longitudinal assessment
Source: PLoS One. 2026 Feb 11;21(2):e0342487. doi: 10.1371/journal.pone.0342487 (PMC12893557; doi:10.1371/journal.pone.0342487)
Supplement: Attachment — Submitted filename: Response_to_Reviewers_auresp_2.docx [file pone.0342487.s006.docx]

**Factors Influencing the Functionality of Medicines and Therapeutic Committees in Public Health Facilities in Uganda: A Longitudinal Assessment**

Benard Nsubuga

National Medical Stores

Plot 261, Kiwamirembe Road, Kajjansi.

**5^th^ January 2026**

**The Editor**

**PLOS One**

**RE: POINT BY POINT RESPONSE FOR THE RESUBMITTED MANUSCRIPT**

Following reviewing of the above manuscript we hereby submit a revised version after addressing the comments from the editor and the reviewer. The table below indicates the editor and reviewers’ comments and the corresponding adjustments that were made in the revised version of the manuscript.

**Editor**

| **Comment** | **Page number** | **Action** |
| --- | --- | --- |
| The manuscript is still very voluminous, and readers may get lost reading through it. The “Methods“ section can still be reduced further. There are also too many tables and figures. Some of these may be moved to “Supplementary materials” section. It is also important to note that not all the results of the study needs to be displayed and discussed - only the relevant ones! | Neutral | Whereas it would be necessary to submit some tables as supplementary/ separate files for purposes of reducing the volume of the manuscript, the author guidelines regarding submission of tables dictate otherwise per the statement below:  “*Place each table in your manuscript file directly after the paragraph in which it is first cited (read order). Do not submit tables in a separate file(s).”*  Additionally, the purpose of this paper was to establish the factors influencing the functionality of MTCs in public health facilities in Uganda. Presentation of findings only retained independent variables that were included at multivariate level. the volume of the findings section could not be reduced any further.  Finally, redundant statements have been dropped from the methods section. Otherwise, the rest of the information especially in the model estimation section provides internal validity to the findings and will be beneficial to advanced readers and will enhance in the literature space. |
| Is the numbering of the sections according to the journal’s format? If not, kindly revise | Neutral | Numbers before the sections/subsections have been removed. |

**Reviewer Comments**

| **Comment** | **Page number** | **Action** |
| --- | --- | --- |
| The authors have significantly improved the Manuscript. However, they should remove the numbers before the subheadings; this is not s thesis. Removing the numbers will improve the manuscript. | Neutral | Numbers before the sections/subsections have been removed. |
| Page 16 mentions a Fig 4: Frequency of MTC meetings in HFs which is not shown in the manuscript, please authors should correct this or delete the mention. | Neutral | Figure 4 was upload together with the other figures |
